# Elexacaftor-Tezacaftor-Ivacaftor: A Life-Changing Triple Combination of CFTR Modulator Drugs for Cystic Fibrosis

**DOI:** 10.3390/ph16030410

**Published:** 2023-03-08

**Authors:** Mafalda Bacalhau, Mariana Camargo, Grace A. V. Magalhães-Ghiotto, Sybelle Drumond, Carlos Henrique M. Castelletti, Miquéias Lopes-Pacheco

**Affiliations:** 1 Biosystems & Integrative Sciences Institute (BioISI), Faculty of Sciences, University of Lisbon, 1749-016 Lisbon, Portugal; 2 Department of Surgery, Division of Urology, Sao Paulo Federal University, Sao Paulo 04039-060, SP, Brazil; 3 Department of Biotechnology, Genetics, and Cell Biology, Biological Sciences Center, State University of Maringa, Maringa 87020-900, PR, Brazil; 4 Center for Research in Bioethics and Social Health, School of Magistracy of the State of Rio de Janeiro, Rio de Janeiro 20010-090, RJ, Brazil; 5 Molecular Prospecting and Bioinformatics Group, Keizo Asami Institute, Federal University of Pernambuco, Recife 50670-901, PE, Brazil

**Keywords:** adverse effects, case report, clinical efficacy, clinical trial, Kaftrio, precision medicine, observational study, real-life study, safety, Trikafta

## Abstract

Cystic fibrosis (CF) is a potentially fatal monogenic disease that causes a progressive multisystemic pathology. Over the last decade, the introduction of CF transmembrane conductance regulator (CFTR) modulator drugs into clinical practice has profoundly modified the lives of many people with CF (PwCF) by targeting the fundamental cause of the disease. These drugs consist of the potentiator ivacaftor (VX-770) and the correctors lumacaftor (VX-809), tezacaftor (VX-661), and elexacaftor (VX-445). In particular, the triple combination of CFTR modulators composed of elexacaftor, tezacaftor, and ivacaftor (ETI) represents a life-changing therapy for the majority of PwCF worldwide. A growing number of clinical studies have demonstrated the safety and efficacy of ETI therapy in both short- and long-term (up to two years of follow-up to date) and its ability to significantly reduce pulmonary and gastrointestinal manifestations, sweat chloride concentration, exocrine pancreatic dysfunction, and infertility/subfertility, among other disease signs and symptoms. Nevertheless, ETI therapy-related adverse effects have also been reported, and close monitoring by a multidisciplinary healthcare team remains vital. This review aims to address and discuss the major therapeutic benefits and adverse effects reported by the clinical use of ETI therapy for PwCF.

## 1. Introduction

**Figure 1:**
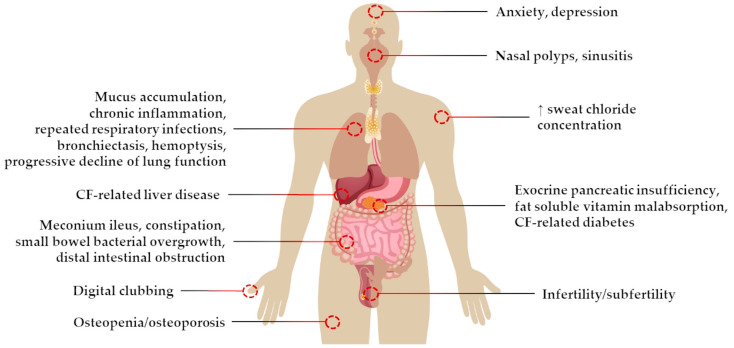
Major clinical manifestations developed by people with cystic fibrosis.

Cystic fibrosis (CF) is a progressive inherited disease with an autosomal recessive pattern and affects more than 100,000 people worldwide [1,2,3]. The disease occurs due to mutations in the gene located on the long arm of chromosome 7 at position 31.2 (7q31.2) that encodes the CF transmembrane conductance regulator (CFTR) protein [4,5,6]. Under normal physiological conditions, CFTR functions as a cAMP-mediated chloride/bicarbonate channel expressed at the apical plasma membrane of several epithelial tissues, where it plays a critical role in electrolyte and fluid balance, thus controlling the composition and quantity of epithelial secretions [7]. In the case of the mutant CFTR gene, there is an impairment of CFTR protein expression and/or function, causing an abnormal ion transport and dehydration of epithelial surface lining that leads to a cycle of accumulation of thick mucus, chronic inflammation, and recurrent infections, which results in tissue damage and remodeling [7,8]. Clinically, the disease has a multisystemic involvement, including severe pulmonary and gastrointestinal manifestations, high sweat chloride concentration (SCC), exocrine pancreatic insufficiency, male infertility, and digital clubbing, among others (Figure 1). However, the progressive decline of lung function represents the major cause of disability and mortality in CF [7,8,9].

The deletion of a phenylalanine at position 508 (F508del) is by far the most prevalent CF-causing mutation, occurring in 80–85% of CF cases worldwide [2]; however, more than 2100 *CFTR* genetic variants have been reported to date (http://www.genet.sickkids.on.ca/, accessed on 22 December 2022), most being presumably pathogenic and leading to a broad range of phenotypes, from classical CF to mild disease symptoms (https://cftr2.org/, accessed on 22 December 2022). Therefore, CFTR mutations have been grouped into six functional classes according to the primary CFTR defect, characterized by: (I) no production of the full-length protein, (II) impaired protein folding and trafficking, (III) defective channel gating, (IV) reduced channel conductance, (V) reduced protein production, and (VI) reduced protein stability at the plasma membrane [2,9,10]. Since people with CF (PwCF) may carry different CFTR mutations on the two alleles, thousands of possible combinations of CF genotypes exist (https://cftr2.org/, accessed on 22 December 2022).

Up to 2012, CF therapies were focused on the management of disease signs, symptoms, and complications. These include inhaled and physical therapies and various daily medications, including conventional mucolytics, antibiotics, anti-inflammatory agents, and pancreatic enzymes, and monitoring by a multidisciplinary healthcare team (physicians, nurse, nutritionist, physiotherapist, and psychologist) [1,3,11]. With the advances in fundamental and translational research by developing preclinical cell models and by implementing cell-based high-throughput screening assays, novel therapies that directly target the primary CFTR defect(s) were discovered [10,12]. These are CFTR modulator drugs that can restore the folding and trafficking of mutant CFTR protein (correctors) or enhance the channel open probability (potentiators) when the protein is located at the plasma membrane [2,11]. To date, four CFTR modulators drugs have been approved for clinical use by both the Food and Drug Administration (FDA) and the European Medicines Agency (EMA): the potentiator ivacaftor (IVA, VX-770) and the correctors lumacaftor (LUMA, VX-809), tezacaftor (TEZA, VX-661), and elexacaftor (ELEXA, VX-445) [13,14,15,16,17,18].

A cutting-edge triple combination drug—composed of ELEXA-TEZA-IVA (ETI)—was approved in 2019 for the treatment of PwCF carrying one or two copies of the F508del mutation [17,18]. Indeed, it has been proposed that ETI may deeply change the disease course in several different manners [2,19]. For instance, it is estimated that the introduction of ETI into clinical practice will increase the life expectancy of PwCF to above 50 years in the United States and the United Kingdom [20,21], but it remains much lower in countries where ETI is still not available [22,23]. Due to the remarkable therapeutic outcomes promoted by ETI, a large number of studies have been pursued to better understand its pulmonary and extra-pulmonary effects in the short- and long-term as well as to expand its label to rarer CF genotypes. This review aims to discuss and aggregate in one piece the major benefits and adverse effects that have been reported to date by the treatment of PwCF with ETI.

## 2. Clinical Trials: From Monotherapy to Triple Combination Therapy

Numerous clinical trials were performed to evaluate the safety and efficacy of IVA (tradename: Kalydeco^®^, Vertex Pharmaceuticals, Boston, MA, USA), LUMA-IVA (tradename: Orkambi^®^, Vertex Pharmaceuticals, Boston, MA, USA), and TEZA-IVA (tradename: Symdeko^®^/Symkevi^®^, Vertex Pharmaceuticals, Boston, MA, USA) in individuals carrying common and rare CF genotypes as well as to expand their clinical use for younger children. The main characteristics of selected clinical trials are reported in Table 1 and further described below. These studies were fundamental for the subsequent clinical development of ETI therapy.

### 2.1. Ivacaftor Monotherapy

Ivacaftor was the first CFTR potentiator evaluated in phase 3 clinical trials that showed significant improvements in the lung function and nutritional status of PwCF carrying at least one G551D-CFTR [13,24,25]. A 48-week randomized, double-blind, placebo-controlled phase 3 clinical trial (termed STRIVE) evaluated IVA monotherapy in PwCF ≥12 years old carrying at least one G551D-CFTR, and a baseline percent predicted forced expiratory volume in one second (ppFEV_1_) of 40–90% [13]. In the cohort receiving IVA, PwCF demonstrated an improvement in ppFEV_1_ and weight as well as a reduction in SCC and the risk of pulmonary exacerbations. Compared to those in the placebo cohort, PwCF receiving IVA also exhibited a higher score on the respiratory-symptoms domain of the Cystic Fibrosis Questionnaire-revised (CFQ-R) instrument (patient-reported respiratory symptoms). Furthermore, the incidence of adverse effects was similar between both cohorts [13].

The following 48-week randomized, double-blind, placebo-controlled trial (termed ENVISION) examined IVA monotherapy in children with CF aged 6–11 years with a G551D-CFTR on at least one allele [24]. In this clinical trial, children receiving IVA monotherapy also showed an improvement in lung function, weight, CFTR activity, and patient-reported respiratory symptoms assessed by a child version of the CFQ-R score. Only one child in the placebo group withdrew from the ENVISION study due to anxiety and psychological issues, and no significant differences in adverse events were verified between the two cohorts [24]. A 24-week, open-label phase 3 trial (termed KIWI) was performed in children with CF aged 2–5 years with at least one CFTR gating mutation to investigate IVA pharmacokinetics, safety, and efficacy [25]. The results of the KIWI study were similar to those performed in older patients. Since its approval by the FDA and the EMA, IVA monotherapy has been used in clinical practice for PwCF carrying G551D-CFTR mutation and for other gating mutations [28]. IVA monotherapy was also approved by the FDA for various CFTR residual function mutations based on in vitro studies [10,29], with clinical benefits being confirmed in subsequent clinical studies for several of these mutations [30,31].

### 2.2. Lumacaftor-Ivacaftor Therapy

Two 24-week-randomized control phase 3 trials (termed TRAFFIC and TRANSPORT) investigated the effects of LUMA plus IVA in PwCF ≥12 years old homozygous for F508del-CFTR [14]. Overall, PwCF treated with LUMA-IVA showed modest improvements in ppFEV_1_ (3–4%), body mass index (BMI), CFQ-R score, and reduction in the rate of pulmonary exacerbations when compared to the placebo cohort. However, the proportion of PwCF who discontinued the study was higher in the treatment groups than in the placebo group (4.2% in the LUMA-IVA groups vs. 1.6% in the placebo group). The adverse events related to the discontinuation of the study in LUMA-IVA groups were the elevation of the creatine kinase levels in four patients, hemoptysis in three patients, bronchospasm in two patients, dyspnea in two participants, pulmonary exacerbation in two participants, and rash also in two participants. Moreover, serious adverse events related to abnormal liver function were associated with the active-treatment group in seven patients [14]. Following this study, a phase 3, double-blind, placebo-controlled trial was performed in PwCF homozygous for F508del-CFTR with ages between 6–11 years [26]. In this study, the primary endpoint was the mean absolute change in lung clearance index_2.5_ (LCI_2.5_) from baseline through the study and up to week 24. The primary endpoint and the absolute change in ppFEV_1_ averaged through week 24 showed a significant difference between LUMA-IVA and placebo groups, and significant reductions were observed in SCC at day 15 and up to week 24 [26]. However, the comparison between the two groups for BMI and CFQ-R respiratory domain scores was not statistically significant. Although the proportion of PwCF reporting adverse events was similar between the two groups, some events were more frequent in the treatment group. Abnormal breathing and elevated aminotransferases were adverse events that led to treatment discontinuation [26]. Overall, despite the fact that this combination therapy showed efficacy in some outcomes, its use has been limited due to some concerns regarding the safety and drug–drug interactions.

### 2.3. Tezacaftor-Ivacaftor Therapy

A randomized, placebo-controlled, double-blind, multicenter, phase 2 study evaluated the safety and efficacy of TEZA, a new corrector, in combination with IVA in PwCF homozygous for F508del-CFTR and compound heterozygous for F508del- and G551D-CFTR [27]. The incidence of adverse events was similar between the cohorts, and most effects were mild to moderate in severity. The incidence of serious adverse events was lower in the active drug arms than in the placebo arm. Overall, TEZA-IVA therapy was well-tolerated with low rates of discontinuation. In PwCF homozygous for F508del-CFTR, the greatest improvements in both SCC and lung function were obtained in the cohort receiving TEZA (100 mg/day) plus IVA (150 mg every 12 h) [27]. In comparison with PwCF treated with LUMA-IVA in the TRAFFIC and TRANSPORT studies [14], the treatment with TEZA-IVA showed greater safety and improvements in lung function for PwCF homozygous for F508del-CFTR [27]. In compound heterozygous subjects for F508del- and G551D-CFTR, the effects observed in SCC and ppFEV_1_ were greater with the addition of TEZA than the effects observed for IVA monotherapy, indicating the potential of the combined therapy for this CF population [27].

A following randomized, double-blind, multicenter, placebo-controlled, parallel-group phase 3 trial (termed EVOLVE) assessed the efficacy and safety of TEZA-IVA in PwCF ≥12 years old who were homozygous for F508del-CFTR [15]. The incidence of adverse events was similar between the two groups, and serious adverse events were less frequent in the active-treatment group than in the placebo. TEZA-IVA therapy led to a significant improvement in the absolute change from baseline in the ppFEV_1_ in comparison to placebo over the study period. Similarly, the pulmonary exacerbation rate was significantly lower in the treatment cohort compared to the placebo [15]. In parallel, a randomized, multicenter, double-blind, placebo-controlled phase 3 trial (termed EXPAND) was designed to evaluate TEZA-IVA in PwCF ≥12 years old who were heterozygous for F508del-CFTR and a second allele with a CFTR mutation with residual function [16]. Treatment showed significant benefits concerning the absolute change in the ppFEV_1_ compared to the placebo cohort. Additionally, TEZA-IVA therapy resulted in significant benefits when compared to IVA monotherapy. Moreover, serious respiratory adverse events related to therapy or discontinuation were not reported over the study period [16]. Similar to IVA monotherapy, label extensions have been approved by the FDA for other CFTR mutations based on in vitro studies [10,29].

### 2.4. Triple Combination Therapy

Despite the advances in CF therapies, the TEZA-IVA combination demonstrated only modest therapeutic effects in PwCF homozygous for F508del-CFTR (similar to LUMA-IVA combination [14,15]) or heterozygous with a second mutation resulting from CFTR residual function [16]. Moreover, clinical benefits were even lower in PwCF heterozygous for F508del-CFTR and a minimal function (MF) mutation on the second allele [32]. Two novel correctors—VX-659 and elexacaftor—were demonstrated to rescue F508del-CFTR folding and traffic to the plasma membrane by different mechanisms of action from those exerted by LUMA and TEZA [33,34]. Accordingly, they were further studied in phase 2 and 3 trials in triple combinations with TEZA-IVA (Table 2).

A randomized, parallel-track, placebo- or active-controlled, double-blind, multicenter, dose-ranging, phase 2 trial was conducted to evaluate VX-659-TEZA-IVA combination in PwCF who were heterozygous for F508del-CFTR and an MF CFTR mutation (F508del/MF genotypes) or homozygous for F508del-CFTR [33]. Most adverse events were mild or moderate, and none led to the discontinuation of the study. The different doses of VX-659-TEZA-IVA resulted in significant improvement in the absolute change from baseline in the ppFEV_1_ in PwCF F508del/MF genotypes and in PwCF homozygous for F508del-CFTR already receiving TEZA-IVA. The SCC and the CFQ-R score improved in both cohorts receiving VX-659-TEZA-IVA compared to the placebo. Overall, the VX-659-TEZA-IVA combination showed additive therapeutic responses in comparison to either placebo or TEZA-IVA therapy in all clinical parameters evaluated [33].

In another randomized, placebo-controlled, double-blind, dose-ranging, phase 2 trial, ELEXA-TEZA-IVA (ETI) therapy was evaluated in PwCF F508del/MF genotypes and PwCF homozygous for F508del-CFTR [34]. The primary endpoints investigated were safety and absolute change in ppFEV_1_ from baseline. Three patients in the active-treatment group and one patient in the control group discontinued treatment because of adverse events. However, most adverse effects were mild or moderate, and the most common included cough, increased sputum production, infective pulmonary exacerbation, hemoptysis, and pyrexia. Regarding efficacy, significant improvements over baseline in the ppFEV_1_ were measured for all administered doses and in both populations treated with ETI. The evaluation of secondary endpoints in both CF genotypes showed improvements in SCC and the CFQ-R score [34]. Overall, ETI therapy presented an acceptable safety and adverse effect profile, similar to that observed for the combination of VX-659-TEZA-IVA [33], and improved lung function in PwCF F508del/MF genotypes who had not been treated with previous CFTR modulators, and in F508del-homozygous PwCF who had previously received TEZA-IVA therapy [34].

Since ELEXA was found to have more suitable pharmacological properties and safety profile for the long term, it was further investigated in phase 3 clinical trials. In a multicenter, randomized, double-blind, active-controlled phase 3 trial, the efficacy and safety of the ETI combination regimen were evaluated for 4 weeks in PwCF homozygous for F508del-CFTR [17]. The triple combination therapy was demonstrated to be superior compared to TEZA-IVA therapy, leading to faster and higher improvement in ppFEV_1_ above the baseline, in SCC, and the CFQ-R score. Furthermore, there was a reduction of infective pulmonary exacerbations in the ETI cohort and no adverse events that led to the discontinuation of the trial [17]. In parallel, another multicenter, randomized, double-blind, placebo-controlled phase 3 trial was conducted for 24 weeks to assess ETI therapy in PwCF ≥12 years old and with F508del/MF genotypes [18]. The triple combination regimen resulted in significant improvement in the primary endpoint of absolute change in ppFEV_1_ at week 4 through week 24, and most adverse events were mild or moderate. Additionally, ETI therapy resulted in a significant reduction of SCC through week 24 and in a 63% lower annualized rate of pulmonary exacerbations and hospitalization compared to the placebo group [18]. Overall, both phase 3 clinical trials confirmed the efficacy of ETI therapy for PwCF carrying F508del-CFTR on at least one allele, which resulted in its approval by both the FDA (tradename: Trikafta^®^, Vertex Pharmaceuticals, Boston, MA, USA) and the EMA (tradename: Kaftrio^®^).

The efficacy of ETI therapy was also assessed for PwCF heterozygous for F508del-CFTR with either a gating mutation or a residual function mutation on the second allele in a multicenter, active-controlled, 8-week phase 3 trial [36]. Based on the genotype, a 4-week run-in period of therapy with either IVA monotherapy or TEZA-IVA was performed, and then the participants were randomly allocated to continue the treatment as controls or to receive the triple combination therapy for 8 weeks. Results showed that those who received the triple combination therapy had a 3.7% improvement in ppFEV_1_ relative to baseline, and further 3.5% relative to active control (i.e., treated before with IVA or TEZA-IVA), and a reduction of 22.3 mmol/L in SCC relative to baseline, and 23.1 mmol/L compared those who maintained the previous therapy [36].

The durability of the clinical effects of ETI has been assessed in a multicenter, randomized, double-blind, and active-controlled phase 3b trial with PwCF homozygous for F508del-CFTR and aged ≥12 years [38]. After a 4-week run-in period of TEZA-IVA therapy was completed, participants were randomly assigned to the active control group (TEZA-IVA) or the cohort receiving the triple combination therapy for an additional period of 24 weeks. Compared with the active control group, the triple combination therapy led to significant improvements in the CFQ-R score (primary endpoint), and in ppFEV_1_ and SCC (secondary endpoints). A total of 89% of participants in the triple combination therapy cohort and 92% of participants in the active control group presented adverse effects that were mild or moderate in severity. One participant in the triple combination therapy cohort and two participants in the active control group withdrew due to psychiatric disorders that were not related to the study [38]. Furthermore, preliminary results of an open-label extension, phase 3 study demonstrated the safety and sustained efficacy of long-term (at least 24 weeks) triple combination therapy in PwCF ≥12 years old and with at least one F508del-CFTR allele [37].

A subsequent 24-week open-label phase 3 trial was performed to assess the safety, pharmacokinetics, and efficacy of ETI therapy in children aged 6–11 years old and with F508del/MF or F508del/F508del genotypes [35]. Safety and tolerability profiles were similar to those observed in older PwCF receiving the triple combination therapy. The most common adverse events included cough, headache, and pyrexia. Regarding efficacy, the triple combination therapy improved ppFEV_1_ through week 24, LCI_2.5_, CFQ-R score, and BMI, and reduced SCC when compared to the pre-treatment baseline [35]. In parallel, a 24-week randomized, placebo-controlled, double-blind phase 3b trial was conducted to evaluate the efficacy and safety of ETI therapy in children aged 6–11 years old and heterozygous for F508del-CFTR and an MF mutation on the second allele [39]. The triple combination therapy led to improvements in absolute change in LCI_2.5_ from baseline through week 24 (primary endpoint), in respiratory symptoms and CFTR function. Furthermore, differences between therapy and placebo cohorts showed significant improvements in lung function. Most adverse events were mild or moderate in severity, and the most common adverse events in the therapy cohort were headache and cough, and in the placebo group were cough, abdominal pain, CF infective pulmonary exacerbation, headache, and oropharyngeal pain. One child discontinued the therapy due to a rash that resolved after discontinuation. No new safety concerns were observed, and results provided further evidence that the triple combination therapy substantially improved CFTR function and clinical outcomes [39]. Overall, these results support the extend of ETI therapy for a younger CF population.

Nichols and collaborators performed a prospective, observational post-approval study (PROMISE) to understand the broad effects of ETI therapy in a diverse CF population in the United States in a 6-month follow-up period [40]. Participants enrolled were aged ≤12 years old (mean age of 25 years) and carrying at least one copy of F508del-CFTR (48% were homozygous for F508del-CFTR). They also completed a baseline study visit before starting ETI: 50.9% were naïve for modulators, 44.1% were on a two-drug CFTR modulator combination, and 6.7% were on IVA monotherapy. Overall, ETI therapy resulted in an improved ppFEV_1_ in the entire cohort, mainly in those previously naïve to modulators or using a two-drug combination. Similarly, the average SCC was greater among these two subgroups, and BMI and the CFQ-R score improved significantly in the entire cohort. The greater improvements in clinical outcomes provided by ETI support the prescription of this therapy not only for PwCF naïve to modulators but also for those already using two-drug combinations or IVA monotherapy [40].

In a recent multicenter, open-label, randomized, controlled clinical study, Mayer-Hamblett and collaborators assessed the possibility of reducing the therapeutic burden in PwCF receiving ETI therapy by removing hypertonic saline or dornase alpha [41]. All participants were receiving ETI therapy and should have received mucoactive therapies (i.e., more than 3% hypertonic saline or dornase alpha) for at least 90 days before the start of this study. The study included 370 individuals using hypertonic saline and 477 using dornase alpha, which were then randomly allocated to continue or discontinue mucoactive therapy for 6 weeks. Overall, the results indicated equivalent clinical outcomes for the continuation or discontinuation of these therapies. In the hypertonic saline cohort, 35% of those who discontinued this therapy had at least one adverse effect, against 24% for those who continued it. In the dornase alpha cohort, 37% of those who discontinued it presented an adverse effect, against 23% for those who continue this therapy. The main adverse effects reported were cough, nasal congestion, chest discomfort, increased sputum production, myalgia, and headache. Despite the significant clinical outcomes observed in this study, it should be kept in mind that these findings were observed in PwCF with relatively well-preserved lung function (ppFEV_1_ ≥60%) and in a short-term (6 weeks) [41].

To date, ETI therapy demonstrated the best clinical efficacy in rescuing F508del-CFTR protein, allowing to target CF sub-populations, namely those carrying two copies of F508del-CFTR or those heterozygous for F508del-CFTR and a gating, residual, or MF CFTR mutation on the second allele. Moreover, a label extension has been granted by the FDA for numerous CFTR mutations based on in vitro studies [10,29].

While this manuscript was in review, clinical results from a novel triple combination of CFTR modulators were published. Uluer and collaborators performed two randomized, double-blind, controlled phase 2 trials to assess the safety and efficacy of vanzacaftor–tezacaftor–deutivacaftor in PwCF aged ≥18 years old carrying F508del-CFTR in at least one allele [42]. Initially, 77 PwCF were assigned into two different groups and received 150 or 250 mg daily of deutivacaftor. Both doses were well-tolerated and safe, and the mean absolute change in ppFEV_1_ was 3.1 points for the lower dose and 2.7 for the higher dose of deutivacaftor. Thereafter, a total of 86 participants (58 with F508del/MF genotype and 28 with F508del/F508del genotype) received different doses of vanzacaftor (5, 10, or 20 mg daily) in combination with tezacaftor and deutivacaftor for four weeks. Three participants discontinued the study due to adverse effects, including cough, headache, increased sputum production, and increased value of liver enzymes. The mean absolute change in ppFEV_1_ was 4.6 points for vanzacaftor 5 mg, 14.2 points for vanzacaftor 10 mg, and 9.8 points for vanzacaftor 20 mg (added to tezacaftor and deutivacaftor triple combination). Moreover, significant improvements in SCC and the CFQ-R respiratory domain score were also reported [42]. Such effects demonstrated the potential of vanzacaftor–tezacaftor–deutivacaftor therapy to provide similar or even greater therapeutic effects than ETI therapy. Phase 3 clinical trials comparing both triple combinations are in progress to confirm such effects (NCT05033080, NCT05076149).

## 3. Clinical Outcomes of ETI Therapy in Case Reports, Observational, and Real-Life Studies

### 3.1. Clinical Studies in PwCF Carrying at Least One F508del-CFTR Allele and Advanced Lung Disease

Numerous clinical studies have been performed to assess the clinical benefits of ETI therapy in PwCF with advanced lung disease (ppFEV_1_ ≤ 40%), since these individuals are usually excluded from initial clinical trials due to their accelerated deterioration of lung function (Table 3).

In a large French cohort, Burgel and collaborators confirmed the effects of ETI therapy in PwCF aged ≥12 years old and advanced lung disease (mean ppFEV_1_ of 29%) with a rapid improvement in ppFEV_1_ soon after the start of ETI therapy [43]. Over the follow-up, there was a reduction in the demand for oxygen therapy (50% reduction), non-invasive ventilation (30% reduction), and enteral feeding. Moreover, most PwCF indicated for transplantation were regressed, denoting a rapid change in the disease course [43]. Carnovale and collaborators also assessed the effects of ETI therapy for one year in 26 PwCF homozygous for F508del-CFTR with advanced lung disease (median age of 31.1 years) [44]. Lung function, nutritional status, SCC, and frequency of pulmonary exacerbations were analyzed. After one year of ETI therapy, the mean absolute improvement in ppFEV_1_ was 14.48 and BMI was 2.08 kg/m^2^. SCC dropped from 77.5 mmol/L to 29.2 mmol/L. The CFQ-R respiratory domain score improved by 32.6 points from baseline, and pulmonary exacerbations were 97% less frequent after ETI therapy [44]. Similar findings were reported in an observational study by O’Shea and collaborators [45]. Fourteen PwCF carrying F508del-CFTR on at least one allele with advanced lung disease were included. All parameters evaluated (ppFEV_1_, BMI, SCC, and frequency of pulmonary exacerbations) improved significantly within the five months of ETI therapy [45].

Terlizzi and collaborators reported the efficacy of ETI in three PwCF carrying F508del-CFTR in one allele and an unknown CFTR mutation on the second allele [46,57]. The three adults had a history of *Pseudomonas aeruginosa* infection, chronic productive cough, and elevated SCC. Before starting the treatment, ETI was tested in nasal epithelial cells collected from these individuals. The results demonstrated that ETI restored CFTR-dependent chloride current in the nasal epithelial cultures, reaching values similar to non-CF nasal epithelial samples [57]. Following the in vitro results, the three individuals started ETI therapy, and clinical data were collected at baseline and 8, 12, and 24 weeks after starting the treatment [46]. The clinical results demonstrated a progressive decrease in SCC in the three individuals, reaching values within the normal range at 24 weeks. The ppFEV_1_ had a relevant improvement from 29.0% to 53.6% in individual #3. The BMI and the CFQ-R score also improved within six months of ETI therapy. Moreover, individuals #2 and #3 discontinued oxygen therapy, while the daily insulin dose of individual #1 was reduced to half after the first 4 weeks of ETI therapy [46]. In the first six months of follow-up, the three individuals did not need antibiotic therapy [46] with clinical outcomes comparable to those obtained by Middleton and collaborators [18].

Another case report demonstrated the efficacy of ETI in PwCF (F508del/MF genotype) with advanced lung disease [47]. In this study, 47 PwCF (20 males, 42.5%) were recruited, and the median (range) age was 30.5 years (17–57.6 years). All patients had pancreatic insufficiency and 25 (53.2%) individuals had CF-related diabetes (CFRD). The results demonstrated an improvement of ~15% in the absolute change of lung function, and SCC decreased to half (from 91.1 to 46.2 mmol/L) after six months of ETI therapy. ETI therapy was also associated with a significant reduction in the incidence of pulmonary exacerbations, the need for oral and intravenous antibiotic therapy, and an improvement in the 6-min walking test distance. Similar findings were also reported in a case report of a 50-year-old woman with CF (F508del/G1244E genotype) [48]. Another case reported the ability of ETI in combating persistent infection in a 24-year-old woman with CF (F508del/R1066C genotype) and advanced lung disease [49]. After 12 years of failed eradication of *Mycobacterium abscessus*, despite numerous combination treatments, ETI therapy led to the eradication of the pathogen and improvement in lung function and nutritional status, resulting in the withdrawal of this individual from the lung transplant list [49].

In a case-control study, the effects of ETI therapy in 26 PwCF (F508del/MF genotype) were followed for one year [50]. The control group was composed of PwCF with mild lung disease, while the study group consisted of PwCF with advanced lung disease. A decrease in SCC in 77% of the participants in the study group was also observed. These findings were accompanied by a decreased rate of microbial colonization, with 45.3% of the samples becoming negative within one year of ETI therapy. When the CFQ-R was evaluated, all PwCF showed remarkable improvement in quality of life with a score of 100—the highest score for this questionnaire [50]. In both groups, ETI therapy resulted in an improvement of 10–15% in ppFEV_1_. Computed tomography (CT) revealed a significant reduction in lung tissue damage with lesser signs of air trapping and disappearing of parenchymal lung nodules. In another study, lung anatomical structure was also evaluated by magnetic resonance imaging (MRI) [51]. Three PwCF (2 females and 1 male) were evaluated 3 months after the start of ETI therapy. They were heterozygous for F508del-CFTR with N1303K-, R553Q-, or L1065P-CFTR on the second allele. The results demonstrated an improvement of ppFEV_1_ as well as in lung structure, assessed by MRI, emphasizing the reduction in bronchial wall thickening and mucus plugging [51].

Stylemans and collaborators performed a real-life, three-months follow-up study and investigated the effects of ETI therapy on LCI and the proximal and peripheral components of the ventilation distribution in 14 adults with CF and advanced lung disease (average age of 36 years, median ppFEV_1_ of 34%) [52]. All these individuals had pancreatic insufficiency, 50% had CFRD, and 64% were homozygous for F508del-CFTR. The LCI (by 31% predicted, below baseline 247% predicted) and ventilation heterogeneity in the acinar compartment (by 411% predicted, below baseline 798% predicted) were measured. Overall, most therapeutic benefits were observed within 2–4 weeks after the start of ETI therapy and particularly beneficial effects were observed in peripheral zones of the lungs [52]. Another prospective, real-life study was performed by Kos and collaborators in a follow-up of 12 months [53]. Changes in ppFEV_1_, BMI, frequency of pulmonary exacerbations, and adverse effects were assessed in 20 PwCF carrying at least one copy of F508del-CFTR and advanced lung disease. Compared to baseline values, there were significant improvements at 12 months of ETI therapy in ppFEV_1_ (mean increase of 13.7%), BMI (mean increase of 1.87 kg/m^2^), CFQ-R score (mean increase of 32.3 points), and frequency of exacerbations (mean of 2.89 vs. 0.19, before and after ETI, respectively). Despite 55% of participants experiencing at least one therapy-related adverse event, these were mild to moderate in severity and no discontinuation of ETI therapy was required [53].

Since ~1% of children with CF aged 6–11 years develop advanced lung disease when standard therapies are inefficient, Salvatore and collaborators investigated the effects of ETI therapy on this population [54]. They followed for 24 months nine children with CF (median age of 9.75 years) who had advanced lung disease and pancreatic insufficiency but were still not on the transplant waiting list or needed oxygen support. Over the follow-up of ETI therapy, there was a significant improvement in the mean of ppFEV_1_ from 31.1% to 53.7%, CFQ-R score from 25 to 100 points, and SCC from 102.7 mmol/L to 23.8 mmol/L. No hospitalization or adverse effects were reported over this period, and only one child had to use antibiotics [54].

McCoy and collaborators retrospectively assessed various clinical parameters, such as ppFEV_1_, BMI, SCC, and QoL, in a two-year post-ETI period in 18 PwCF (aged 15–49 years) with advanced lung disease [56]. Overall, ETI therapy was well-tolerated, and no participant discontinued it in the two-year follow-up period. There were no new safety concerns, and no participant required a lung transplant over the follow-up period. Regarding efficacy, data demonstrated a sustained improvement in clinical outcomes, including lung function, nutritional status, and respiratory symptoms, which resulted in a reduction in therapy burden and, consequently, a better quality of life for PwCF [56].

Taken together, these studies provide strong evidence of the therapeutic effects of ETI for PwCF who have at least one copy of F508del-CFTR and advanced lung disease, indicating that this CF population can benefit clinically from this therapy.

### 3.2. Case Reports in Rare (Non-F508del) CF Genotypes

As described above, the FDA has granted a label extension for the treatment with ETI to almost 200 CFTR mutations based on in vitro studies [10,29]. Various assays using samples from PwCF have been developed to provide a more reliable prediction of drug effectiveness at an individual level. These include the use of primary bronchial/nasal epithelial cells and rectal biopsies to generate intestinal organoids, and they have supported a translational perspective to include rare CF genotypes that are responsive to ETI therapy [2,12].

For instance, laboratory data demonstrated that ETI can rescue N1303K-CFTR processing and function in heterologous expression systems [58]. Based on these findings and after insurance approval, Huang and collaborators started to treat an 11-year-old girl (N1303K/E193X genotype) with ETI. She had a history of chronic cough with recurrent sinusitis, exocrine pancreatic insufficiency, and low BMI. Over 10 months of ETI therapy, her clinical features improved significantly with a reduction in cough and sinus symptoms and an increase in appetite and energy [58]. In another study, Elson and collaborators reported a case of a 15-year-old boy who was diagnosed with CF (N1303K/Q493X) at birth due to meconium ileus [59]. This individual presented mild lung disease, pancreatic insufficiency, and non-tuberculous *M. abscessus* infection. Due to some preliminary in vitro evidence of the efficacy of TEZA-IVA to rescue N1303K-CFTR, this individual started to be treated with this combination, but his SCC remained elevated in the 12-month follow-up. His therapy was thus changed after the approval of ETI, and he demonstrated an increase in weight (+2.9 kg) and ppFEV_1_ (from 84.3% to 93.3%) within the first six weeks of the new therapeutic regimen. The CFQ-R score also revealed improvements in physical, vitality, and health domains [59].

Burgel and collaborators used a French compassionate program to assess for 4–6 weeks the effectiveness of ETI therapy in 84 PwCF (16 children and 68 adults) with advanced lung disease and carrying no F508del-CFTR in their alleles [60]. Among the participants, 23 had at least one FDA-approved CFTR variant and 61 had none. At 4–6 weeks, 54% of participants (of which 23 had at least one FDA-approved CFTR variant and 22 had none) responded positively to ETI therapy and were able to continue it after the trial period. The remaining 46% of participants demonstrated no clinical improvement with ETI and discontinued the therapy after the follow-up period. Among 54% of participants that were considered to be responders, SCC decreased by 30 mmol/L, ppFEV1 increased by 10.0 percentage points, and body weight increased by 1.7 kg. Such findings hold clinical relevance since 22 participants that were responsive to ETI therapy had rare CFTR variants that are included in the label extension granted by the FDA. In agreement with these results, the authors suggested that an evaluation of ETI effectiveness in PwCF on an individual level could reduce the number of PwCF who still lack eligibility [60].

Stekolchik and collaborators reported a case of a 16-year-old girl (G85E/G85E genotype) who was diagnosed at birth due to meconium ileus [61]. She had pancreatic insufficiency and CFRD. One month after the start of ETI therapy, she presented a complete resolution of respiratory symptoms, and her BMI improved, although CFRD remained difficult to be controlled [61]. In a retrospective study, Livnat and collaborators analyzed data from PwCF without an F508del-CFTR allele and treated with ETI for six months [62]. Data included were lung function, SCC, nutritional status, frequency of pulmonary exacerbations, and antibiotic therapy. Among the 16 PwCF, 8 did not receive any modulator before ETI therapy, and these demonstrated a decrease in the mean of SCC (from 113 to 64 mEq/L) frequency of pulmonary exacerbations (from 1.5 to 0) total antibiotic days per year (from 36 to 0), while the mean of ppFEV_1_ increased (from 66.3% to 72.4%) after ETI therapy. Weight changes were not statistically significant. Five individuals had a G85E-CFTR on at least one allele, indicating clinical evidence of ETI therapy for this mutation. On the other hand, those individuals who were previously treated with other CFTR modulators demonstrated no significant evidence of clinical improvements.

Mitropoulou and collaborators reported a case in which intestinal organoids from a 19-year-old woman (1677delA/R334W) were used to select which clinically approved modulator therapy could be suitable for this individual [63]. Organoids were treated with IVA, LUMA-IVA, TEZA-IVA, and ETI, and an increase in forskolin-induced swelling values was observed with the different modulator combinations but to a comparable extent to that of IVA monotherapy. These findings suggest that modulator combinations would provide similar clinical benefits to that of IVA monotherapy to this individual. Accordingly, she started to receive IVA monotherapy and exhibited a significant improvement in ppFEV1 (from 61% to 99%) and SCC (−26 mmol/L) within 9 months of therapy [63].

Even though there are still few studies in PwCF with non-F508del genotypes, more studies are expected to be performed to assess the clinical benefits of ETI therapy for rare CF genotypes. Moreover, samples from PwCF represent valuable tools to comparatively assess drug efficacies in vitro in order to predict the magnitude of responsiveness and select which therapy may provide the best therapeutic outcomes for each person.

### 3.3. Additional Respiratory Implications

As the progressive deterioration of lung function represents the major cause of morbidity and mortality in CF, various studies have been focused on further understanding the clinical implications of ETI therapy in the different aspects of the disease in both the upper and lower respiratory tract (Table 4).

Data from 43 adults with CF carrying at least one F508del-CFTR allele were included in a retrospective, observational study to evaluate chest MRI scores and chronic rhinosinusitis–MRI scores before and after the start of ETI therapy [64]. Nineteen individuals were receiving ETI therapy, while 24 individuals were stable controls and received only standard treatment without CFTR modulators. Data from the baseline and after a median of 27 months of ETI therapy were assessed. PwCF receiving ETI therapy demonstrated a reduction in the MRI global score and improved MRI morphology, mainly related to the reduction of bronchiectasis, wall thickening, and mucus obstruction sub-scores. There was also an improvement of 6.1 ppFEV_1_ between the two MRIs (before and after treatment) in PwCF receiving ETI, although there was no regression in perfusion abnormalities. Paranasal sinus MRI also indicated a reduction in symptoms due to decreased abnormalities in mucopyoceles in the maxillary and ethmoid sinuses by ETI therapy. Overall, results from MRI demonstrated the reversibility of abnormalities in the paranasal sinus and lung structures by ETI therapy, which correlated with the improvements observed in spirometry [64]. Streibel and collaborators also assessed the effects of ETI therapy on functional and structural lung abnormalities by MRI in 24 children with CF with at least one F508del-CFTR allele [66]. Clinical data, lung function, and MRI were collected one year before the start of ETI, soon after its start, and one year after it. Lung function and BMI improved, while the frequency of acute exacerbations decreased after the start of ETI therapy. A correlation between all lung function parameters and MRI outcomes was observed [66]. Another observational study was performed by Stapleton and collaborators to assess the effects of ETI therapy on sinonasal symptoms of 34 adults with CF (average age of 27 years, 67% homozygous for F508del-CFTR) [67]. Data revealed improved symptoms, such as endoscopic crust, significantly reduced nasal polyps, muconasal thickening, and sinus opacity rapidly and durably for at least 180 days on ETI therapy [67]. Bode and collaborators also reported a reduction in sinonasal symptoms in both children and adults with CF after the start of ETI, besides improved lung function and SCC [77]. Using a different approach, Bec and collaborators aimed to investigate the morphological changes in the respiratory tract of PwCF by analyzing CT images before and 12 months after the start of ETI therapy [69]. Data were collected from 12 individuals with a mean age of 36 years, who had advanced lung disease, and at least one copy of F508del-CFTR (42% homozygous, 58% heterozygous). They found a reduction in lower mucus plugging and peribronchial thickening after ETI therapy. Although there were no significant changes in bronchiectasis, parenchymal injury, and hyperinflation scores, ETI therapy was demonstrated to improve clinical and functional parameters, with an exception for total lung capacity [69], which reinforced the need for an early start of this therapy in order to prevent tissue remodeling.

In a retrospective study, Beck and collaborators assessed the presence of common CF pathogens in samples (mainly sputum) of PwCF receiving ETI therapy for at least one year [79]. Approximately 54%, 33%, and 31% of cultures were positive for *P. aeruginosa,* methicillin-susceptible *Staphylococcus aureus*, and methicillin-resistant *S. aureus* before ETI therapy. A reduction of approximately 30% in the detection of *P. aeruginosa* was noted in bacterial cultures from PwCF after ETI therapy, demonstrating the ability of this therapy in combating persistent infections [79].

Lee and collaborators used the data from the US CF Foundation Patient Registry to investigate the impact of ETI therapy on the rate of lung function decline [68]. To do so, each individual with CF using ETI was score matched with up to five PwCF not using it. The inclusion criteria were PwCF (F508del/F508del or F508del/MF genotypes) aged ≥12 years and with more than three non-missing FEV_1_ measures through a 2-year follow-up. A total of 468 PwCF receiving ETI therapy were included and matched with 1714 untreated controls. In PwCF receiving ETI therapy, there was an increase of 0.39 percentage points in lung function, while it decreased by 1.92 percentage points in the untreated controls, suggesting that ETI therapy has a remarkable effect on preventing an accelerated lung function decline in PwCF [68].

Around 40–60% of PwCF develop rhinonasal signs and symptoms in either childhood or adulthood [80]. In a study performed by DiMango and collaborators [70], 43 adults with CF carrying at least one copy of F508del-CFTR were enrolled to investigate the effects of ETI therapy on sinonasal symptoms. The CFQ-R and sinonasal outcome test (SNOT) questionnaire were also assessed at the baseline and three months after the start of ETI therapy. The initial SNOT demonstrated a reduction from 34.8 to 24.4 with a significant improvement in each of the six domains, and the CFQ-R improved from 60.6 to 83.3 points. Individuals who had been taking another CFTR modulator before the start of ETI therapy demonstrated higher SNOT scores at baseline in comparison to those who were naïve to CFTR modulators [70]. Indeed, a previous study has demonstrated that ivacaftor decreases mucus viscosity and increases ciliary beating and mucociliary clearance [81], which may result in greater performance in the cases in which ETI therapy is demonstrated to be more effective.

Since olfactory disorders are considerably more frequent in PwCF than in age-matched healthy people [82], some studies have investigated the potential impact of ETI therapy on olfaction. In a study performed by Bacon and collaborators, 74% of PwCF had olfactory dysfunction and, despite ETI therapy being demonstrated to improve sinonasal signs and symptoms, there was a lack of improvement in smell discrimination, which might be due to loss of olfactory neurons secondary to chronic inflammation [73]. Similar findings were also reported by other case reports [74,75].

Previous studies have reported the potential effects of CFTR modulators in improving physical activity and exercise tolerance [83,84,85]. Causer and collaborators assessed whether ETI therapy could improve these parameters in three adolescents with CF after six weeks of the start of the therapy [71]. They were homozygous for F508del-CFTR and presented moderate to advanced lung disease before the start of ETI therapy. After the 6-week follow-up, the three participants exhibited an improved peak of the volume of oxygen and exercise capacity, suggesting that ETI therapy might have enhanced the efficiency of respiratory and peripheral muscles [71]. With a similar aim, Giallongo and collaborators assessed cardiorespiratory polygraph parameters and respiratory muscle strength during sleep in nine PwCF before and 12 months after ETI therapy [78]. Although apnea-hypopnea index and oxygen desaturation did not change over the follow-up period, the 6-min walking test, mean expiratory pressure, time spend in oxygen therapy, nocturnal saturation, and mean respiratory rate significantly improved after 3 months of ETI therapy and were sustained throughout the study period [78].

In a study performed by Martin and collaborators, over 100 online questionnaires submitted by adults with CF (mean age 35 years) were used to investigate the impact of ETI therapy from the patient’s point of view [72]. They had advanced lung disease and were on ETI therapy for a mean of 4.3 months. In general, the participants reported significant improvements in respiratory symptoms, including cough reduction and a decrease in sputum production. Most participants indicated being free from exacerbations since the start of ETI therapy and also reported improvements in various extra-pulmonary symptoms [72]. In another qualitative study, interviews were performed to explore the impact of ETI therapy on the lives of children with CF and their attitudes toward nebulization and airway clearance techniques [86]. Overall, ETI therapy was indicated to improve the health of children with CF and reduced the daily treatment burden, despite some concerns about the benefits in the long term.

Altogether, these studies indicate that ETI therapy can reverse several abnormalities in the respiratory tract of PwCF, resulting thus in improved lung function and exercise capacity. However, an early start in life of ETI therapy might be needed to prevent some more persistent abnormalities from occurring, such as bronchiectasis and olfactory disorders.

### 3.4. Pre- and Post-Transplant Implications

Due to the progressive nature of CF, the transplant remains the last resource for those with end-stage disease. A growing number of case reports have investigated the impact of ETI therapy on PwCF who are referred to the transplant list and on those who already underwent liver or lung transplantation (Table 5).

In a study performed by Bermingham and collaborators, 50 PwCF from three CF centers were included [87]. They had advanced lung disease with the need for oxygen therapy, were referred for lung transplantation and pancreatic insufficiency, and were eligible for ETI therapy. Despite the short-term follow-up, from 33 PwCF with transplant referral at the beginning of this study, only 14 remained on the list after 6 months of ETI therapy. The mean of ppFEV_1_ with the start of ETI therapy was 31.6 ± 5.6 to 39.7 ± 10.8 [87]. No differences were observed between PwCF with one or two copies of F508del-CFTR, consistent with the findings reported in other studies [45,88]. In a longer-term follow-up study, Martin and collaborators assessed a French cohort of PwCF referred to lung transplantation for 12 months after the start of ETI therapy [88]. These authors had previously reported a rapid improvement of lung function in PwCF with advanced lung disease after three months of taking ETI [43], but only PwCF referred to lung transplantation were included in this subsequent study [88]. Sixty-five PwCF were included in the study, of which 17 were on the transplant list and 48 were under consideration to be included. After one year of ETI therapy, ppFEV_1_ increased by a mean of 16.3%, and the BMI was +7.4 kg/m^2^. A significant drop in the need for intravenous antibiotics at least once a year (from 93.7% to 36.5%), days of hospitalization, non-invasive ventilation (−62%), long-term oxygen therapy (−59%), and enteral feeding (from 15 to 3 PwCF) was also reported after one year of ETI therapy. Most remarkable improvements were observed within the three first months of ETI therapy and were sustained for the whole period of this study. These findings led to the indication that ETI therapy might reduce the need for lung transplantation due to the significant improvement in clinical outcomes [88].

In lung-transplanted recipients, the potential beneficial effects of ETI have been assessed for other CF-related implications, such as sinus disease, gastrointestinal symptoms, and CFRD [89,90,91]. Benniger and collaborators followed nine adults with CF during one year of ETI therapy and observed an improvement in BMI, gastrointestinal and sinus symptoms, as well as a decrease in glucose levels. One of the participants was also able to discontinue insulin therapy [89]. Nevertheless, another two studies demonstrated a lack of substantial benefit of ETI therapy in lung transplanted recipients, which resulted in an early discontinuation of ETI therapy by many participants. Doligalski and collaborators followed for one year of ETI therapy 14 lung transplant recipients, which started modulators in a median of 115 ± 92 months after the transplant. Over the follow-up, five participants discontinued ETI therapy due to a decline in lung function or mood disturbances. The remaining eight participants who continued ETI therapy demonstrated no differences in lung function and BMI, although improvements in sinus and gastrointestinal symptoms were reported by six and four individuals, respectively. Only one participant demonstrated an improved glycemic index [90]. In the study performed by Ramos and collaborators, only 8 out of 94 lung transplant recipients eligible for ETI therapy received the prescription in the first year after the transplant. No significant differences were observed in lung function (ppFEV_1_ and forced vital capacity) and BMI, although hemoglobin A1c decreased in all participants, with a more meaningful drop in those with CFRD. More than 40% of participants discontinued ETI therapy in a median of 56 days after starting it [91].

Despite some evidence of mild hepatotoxicity of ETI therapy in clinical trials [17,18], the significant improvements in lung function of PwCF led to the investigation of ETI therapy for liver transplant recipients [92,93]. McKinzie and collaborators reported two cases of liver transplant recipients followed up to 20 months after the start of ETI therapy. The first participant demonstrated improved lung function and BMI and the absence of *P. aeruginosa* in respiratory cultures since the start of ETI therapy. An increase in liver enzyme values was observed after one year of ETI therapy but reversed by modulator dosage adjustment [92]. The second participant exhibited preserved lung function at 90% and improved BMI (from 27 to 30.4 kg/m^2^) after three and 20 months of ETI therapy, respectively. However, 8 months following ETI therapy, this individual experienced a mild acute cellular rejection with increased liver enzyme values, which were resolved by corticosteroid therapy. The start of liver rejection was not associated to ETI therapy [92]. Another case series was reported by Ragan and collaborators that followed 10 liver transplant recipients (median age of 22.1 years and started modulators in a median of 6.9 years after the transplant) [93]. Of 10 participants, 8 were using Tacrolimus and two were using Sirolimus, and their dose needed to be adjusted in six participants over the study. In general, ETI therapy was well-tolerated with an increase in liver enzyme values at the start of modulators that was stabilized after a couple of weeks. Most participants demonstrated stable or improved lung function and BMI, symptoms, and quality of life score [93].

Another case report followed four transplant recipients receiving ETI therapy for at least six months [94]. Two underwent bilateral lung transplantation, and two had liver transplantation. Three participants demonstrated a drop in SCC similar to non-transplanted PwCF receiving ETI therapy. Intriguingly, lung function improvements were significantly larger in liver transplant recipients with native lungs, despite all participants exhibiting improvements in respiratory symptoms. Three participants also had a subjective relief of rhinosinusitis symptoms and increased BMI. There was no discontinuation of ETI therapy and no signs of organ rejection, although the dose of Tacrolimus had to be adjusted in two participants during the first six months of the study [94].

Taken together, these studies indicate that ETI therapy may be beneficial for some PwCF who already underwent liver or lung transplantation, thus preventing other CF-related complications, or for those who are referred to the transplant list, thus leading to a significant clinical improvement that no longer would require transplantation. Nevertheless, close therapeutic drug monitoring of these individuals is imperative. Further studies should also be performed to better understand the drug–drug interaction between ETI and immunosuppressives.

### 3.5. Gastrointestinal Implications

CFTR dysfunction results in various detrimental effects on the gastrointestinal tract, and various clinical studies have aimed to further understand the implications of ETI therapy on it (Table 6).

In a multicenter prospective observational study, Schwarzenberg and collaborators investigated the gastrointestinal effects of ETI therapy on PwCF aged ≥12 years and at least one copy of F508del-CFTR [95]. More than 400 PwCF were enrolled in this study and around 50% of the participants received other modulator drugs before ETI. Gastrointestinal effects were assessed by measuring fecal calprotectin, steatocrit, and elastase 1 before and 6 months after the start of ETI therapy as well as three validated questionnaires (Patient Assessment of Upper Gastrointestinal Disorders-Symptom, Patient Assessment of Constipation-Symptom, and Patient Assessment of Constipation-Quality of Life). After 6 months of ETI, fecal calprotectin-1 values decreased, suggesting a decrease in intestinal inflammation. Elastase-1 and steatocrit values nevertheless remained unaltered along the time, suggesting no improvement of pancreatic dysfunction. Gastrointestinal questionnaire scores indicated a decrease in symptoms, although not statistically significant. Overall, 6 months of ETI therapy demonstrated lesser pronounced improvement of gastrointestinal symptoms compared to respiratory symptoms [95].

In a multicenter prospective observational study, Schwarzenberg and collaborators investigated the gastrointestinal effects of ETI therapy on PwCF aged ≥12 years and at least one copy of F508del-CFTR [95]. More than 400 PwCF were enrolled in this study and around 50% of the participants received other modulator drugs before ETI. Gastrointestinal effects were assessed by measuring fecal calprotectin, steatocrit, and elastase 1 before and 6 months after the start of ETI therapy as well as three validated questionnaires (Patient Assessment of Upper Gastrointestinal Disorders-Symptom, Patient Assessment of Constipation-Symptom, and Patient Assessment of Constipation-Quality of Life). After 6 months of ETI, fecal calprotectin-1 values decreased, suggesting a decrease in intestinal inflammation. Elastase-1 and steatocrit values nevertheless remained unaltered along the time, suggesting no improvement of pancreatic dysfunction. Gastrointestinal questionnaire scores indicated a decrease in symptoms, although not statistically significant. Overall, 6 months of ETI therapy demonstrated lesser pronounced improvement of gastrointestinal symptoms compared to respiratory symptoms [95].

A retrospective observational study performed by Petersen and collaborators included data from 134 adults with CF to assess the effects of ETI therapy on body weight and metabolic parameters, such as BMI, systolic and diastolic blood pressure, diabetes, total cholesterol, LDL-c, and HDL-c after a mean of 12 months of therapy [96]. An increase in BMI and body weight was observed with a significant interaction between the effects of ETI on BMI and pancreatic sufficiency status. PwCF with pancreatic insufficiency exhibited a significant difference in BMI trajectory with ETI therapy, while those with pancreatic sufficiency did not. Other changes included an increase in systolic and diastolic blood pressure in the entire cohort, an increase in total cholesterol, HDL-c, and LDL-c in individuals with CFRD, and a decrease in random blood glucose and hemoglobin A1c in individuals without CFRD [96]. In another study, Sully and collaborators aimed to assess the effects of ETI therapy on glycemia in 34 adults with CF using continuous glucose monitoring [97]. Results demonstrated that ETI therapy improved glucose, hyperglycemia, and glycemic variability with the most notable improvements observed in individuals with CFRD. PwCF that had the most expressive improvements in glycemic variability were those that also exhibited the greatest increase in ppFEV_1_ [97]. These findings are also in line with those reported by Korten and collaborators, which verified similar results of ETI therapy in 16 adolescents with CF in an observational pilot study [102]. Steinack and collaborators also reported changes in oral glucose tolerance tests in PwCF with pancreatic insufficiency receiving ETI therapy for at least three months [105]. During the test, glucose levels significantly reduced after 60, 90, and 120 min, indicating an improved glucose tolerance. A decrease in the inflammatory status was observed with a reduction in circulating C-reactive protein, IgG, and IgE levels, which might have an indirect impact on the improved oral glucose tolerance [105]. Aiming to further investigate the effects of ETI therapy on glycemia and pancreatic function, Chan and collaborators collected data from 12 youth and adults with CF before and after at least 8 weeks of the therapy [103]. Participants had a median age of 20.4 years, 75% were men and 90% had pancreatic insufficiency. Despite an increase in BMI, no differences were observed in glucose tolerance before and after the start of ETI therapy. There was also no change in β-cell function adjusted for insulin sensitivity [103]. Due to the controversial findings, further studies in large cohorts are required to better understand the impact of ETI therapy on CFRD.

Because PwCF present poor absorption of lipophilic vitamins, Wright and collaborators investigated the effects of ETI therapy on vitamin D absorption by measuring serum 25-hydroxyvitamin D in 76 participants [98]. Over the study follow-up, 19 individuals required changes in cholecalciferol dose, which was attributed to variations in adherence, formulation, and access to vitamin supplementation. In this group, the median increase in serum vitamin D concentration was 2 ng/mL, while the remaining 57 individuals who did not need a change in cholecalciferol dose exhibited a median increase in serum vitamin D concentration of 5 ng/mL [98]. Such effects may be related to changes in pancreatic absorption or vitamin D processing and use by the body. It has been suggested that such improvements in the long term might lead to a reduction of osteopenia and fracture risk in adults with CF. Moreover, Fancalanci and collaborators reported in a recent study that ETI therapy not only increased BMI and vitamin D absorption but also vitamins A and E absorption in PwCF, demonstrating the ability of this therapy in improving nutritional status [107].

A prospective, longitudinal study performed by Shakir and collaborators assessed the effects of the start of ETI therapy on the symptoms from the upper respiratory and gastrointestinal tract of 32 PwCF [99]. All symptoms related to the sinonasal disease as well as gastroesophageal and extraesophageal reflux significantly improved within the first three months of ETI therapy and were sustained for the 6-month follow-up. It has been suggested that certain improvements in upper gastrointestinal symptoms may be directly related to the improvements in lung health since reflux can be exacerbated by abdominothoracic pressures generated during coughing. Nevertheless, CFTR modulator therapy can improve gastro-duodenal pH, and ETI therapy may thus have a direct effect on esophageal motility and gastric emptying [99]. In a subsequent study, Mainz and collaborators observed similar results in a cohort of 107 PwCF receiving ETI therapy for 26 weeks [104]. A CF-specific abdominal symptoms questionnaire score (CFAbd-Score) was used and 45 healthy individuals were also included for a comparative assessment. The results demonstrated a highly significant direct impact of ETI therapy on self-report abdominal symptoms in PwCF and the mean total CFAbd-Score decreased by 29% over the study follow-up. ETI therapy also significantly reduced abdominal symptoms, regardless of previous therapy with less effective CFTR modulators [104].

In a case series, Safirstein and collaborators described that seven PwCF developed clinically significant biliary colic soon after the start of ETI therapy, and some of them required cholecystectomy [100]. It has been suggested that adults with CF with asymptomatic gallbladder disease may have exacerbated biliary pathology after the start of ETI therapy due to a recovery of CFTR function in the biliary epithelia [100]. Another severe gastrointestinal adverse effect has been reported in a case report of a 21-year-old woman [101]. Before she started ETI therapy, the values for AST, ALT, albumin, and total bilirubin were under the normal range; however, in five months of therapy, there was an increase in the transaminase values, which continued to increase for the subsequent two weeks. Despite ETI therapy leading to an improvement in her lung function (from 35% to 53%), the therapy had to be discontinued due to the inflammatory response from hepatocyte injury. The transaminases of this individual achieved peak values 6 months after the discontinuation of ETI [101], highlighting the need to monitor liver biomarkers until they return to normal ranges.

In a large single-center study, Twekesbury and collaborators reported the effects of ETI therapy on liver tests in PwCF receiving it for over one year [106]. A total of 255 PwCF were enrolled, of which 78 had CF-related liver disease, 20 presented cirrhosis and/or portal hypertension, 4 had a liver transplant and 7 presented isolated portal hypertension or splenomegaly without cirrhosis. Compared to data at baseline, AST, ALT, total bilirubin and albumin values increased in the first 3 months of ETI therapy and were sustained throughout the study period. There were no differences in liver test values between individuals with or without CF-related liver disease [106]. Using a different approach, Wood and collaborators observed a potentially higher incidence of transaminitis in adults with CF receiving ETI, despite it did not result in discontinuation of the therapy [109]. Schnell and collaborators also reported abnormalities in liver stiffness and bile acid metabolism in children and young adults with CF after the start of ETI therapy [108]. Twenty individuals between 10 and 20 years old were followed up for 6 months. Compared to baseline data, improvements in lung function, BMI, and SCC were noted after the start of ETI therapy. Although liver ultrasound did not exhibit changes in size and length, total and direct bilirubin values were significantly increased, highlighting the importance of close monitoring for potential hepatic complications in young PwCF receiving ETI therapy [108].

Taken together, these studies indicate that PwCF receiving ETI therapy should be closely monitored due to potential alterations in liver enzymes, over-nutrition, and related complications. Further studies are thus needed to better understand the long-term clinical effects of ETI therapy on the gastrointestinal tract.

### 3.6. Fertility and Pregnancy Implications

ETI therapy has been associated not only with improved clinical outcomes in PwCF but also with increased CF women’s fertility. Accordingly, a growing number of reports have been performed to assess the effects of ETI therapy on embryonic and fetal development during pregnancy and babyhood (Table 7). Such information is fundamental to optimizing care for PwCF and improving the know-how in family planning and childbearing for CF women receiving ETI therapy [110].

O’Connor and collaborators performed a retrospective study in two centers and identified 201 CF women with infertility/subfertility, of which 14 got pregnant during the first 8 weeks after the start of ETI therapy [111]. Among those, four women had a history of infertility, and two performed in vitro fertilization with no success. Although the mechanism of action is not completely elucidated, it has been suggested that ETI therapy might lessen factors that affect fertility, such as uterine mucus [111]. An increase in pregnancy was also observed in women with CF carrying G551D-CFTR after the start of IVA monotherapy [118].

In another study, Chamagne and collaborators compared two pregnancies of a woman with CF receiving or not ETI therapy in each case [112]. During the first pregnancy, she was 32 years old, not using ETI therapy, and had chronic infection with *P. aeruginosa* and methicillin-resistant *S. aureus*, pancreatic insufficiency, and CFRD. She got pregnant after 7 years of different in vitro fertilization cycles. The pregnancy had no intercurrences, and the baby presented no abnormalities. Over the pregnancy period, she gained only 3 kg, had two hospitalizations with antibiotic therapy, and had three exacerbations with the need for nocturnal oxygen therapy. There was a decrease in ppFEV_1_ (from 50% to 31%) and HbA1c (from 6.6% to 5.7%). The delivery was planned for the 33rd week due to concerns with this individual’s health. The baby was born healthy, and they were able to return home 22 days after the delivery. In the second pregnancy, she was receiving ETI therapy and became spontaneously pregnant 20 days after the start of the therapy. The physicians agreed to continue ETI therapy during the pregnancy due to the clinical benefits for this individual and the unknown risks to the baby. Enteral feeding and chest physiotherapy were discontinued. She gained 10 kg and her ppFEV_1_ was from 53% to 60%. HbA1c remained at 5.5% before conception and continued until delivery. No hospitalizations were required, and only one exacerbation occurred during the pregnancy period. There was a membrane rupture at 34.5 weeks of gestation and a caesarian section was performed. They were able to return home 11 days after childbirth [112].

Collins and collaborators assessed ETI exposure to infants to mothers receiving modulators [113]. Maternal and infant blood, cord blood, and breast milk were collected from three mother–infant pairs from women who received ETI therapy while pregnant. Results demonstrated a relatively high concentration of these drugs in cord blood and low levels in breast milk and infant blood. These findings indicate that ETI can be transferred via the placenta and by breastfeeding in the pre- and post-natal periods, respectively. Nevertheless, the fetal drug concentrations are expected to be within the therapeutic range since ETI concentrations in cord blood were higher or similar to those in the maternal serum. Furthermore, TEZA concentration was higher in breast milk than in the maternal serum, suggesting that this compound may have greater affinity than the others to accumulate in breast milk, which may also explain its relatively higher levels than the other drugs in the infant serum. One of the infants was diagnosed with CF during the study follow-up. She was born with normal pancreatic function, but the elevated lipase concentration at her 9-month life could indicate a slow development of pancreatic dysfunction associated with decreased exposure to ETI over time [113].

Patel and collaborators assessed the levels of immunoreactive trypsinogen in newborns from mothers with CF receiving ETI therapy [119]. The study was performed between 1 January 2020 and 2 June 2022. A total of 636 newborns presented increased levels of immunoreactive trypsinogen and had a CFTR mutation detected in at least one allele. Of these, 51 newborns were diagnosed with CF, 21 had CFTR-related metabolic syndrome, and 489 were CF carriers (66% had an F508del-CFTR). In comparison to ETI-exposed newborns, all these groups exhibited higher mean levels of immunoreactive trypsinogen: 191.6 ng/mL in newborns with CF, 96.7 ng/mL in those with CFTR-related metabolic syndrome, and 66.9 ng/mL in CF carriers. These findings raised some concerns about false negative results of immunoreactive trypsinogen in newborns with CF from mothers receiving ETI therapy [119]. In another study, Fortner and collaborators reported a case of a woman that became pregnant soon after she started receiving ETI therapy [114]. From this pregnancy, an infant with CF (F508del/F508del genotype) was born without any apparent organ dysfunction typically seen in the first days of the life of CF cases. The immunoreactive trypsinogen was normal, which led to a false-negative screen test result. Over the follow-up of this study, high SCC was the only clinical feature observed in this infant with CF [114]. Another woman with CF (30 years old, F508del/Y1092X) got pregnant four months after she started ETI therapy [115]. Her FEV_1_ improved one month after the start of ETI therapy and remained between 35–40% throughout the pregnancy. Her nutritional status also improved, and she did not develop gestational diabetes. No complications occurred up to the 31st week of pregnancy when she presented symptoms of threatened preterm labor. Meanwhile, a rhinovirus infection required the need for oxygen supplementation during the sleep period and the use of inhaled formoterol/budesonide. She gave birth to a healthy infant through vaginal delivery on the 36th week of pregnancy. After childbirth, oxygen therapy was stopped and her FEV_1_ improved again to 40% of the predicted value. Over the follow-up of one year, no further complications were reported [115]. In another case report performed by Szentpetery and collaborators, the effects of ETI therapy were assessed on a woman (F508del-CFTR carrier) who was pregnant with a fetus with CF (F508del/F508del genotype) [116]. At 23 weeks’ gestation, meconium ileus was evidenced by ultrasound imaging with delated hyperechoic bowel, which persisted on subsequent imaging. The woman started ETI therapy at 32 weeks of pregnancy aiming to treat the fetal meconium ileus, which resulted in reversion of bowel dilation by day 27. At 36 weeks gestation, a female infant was delivered vaginally with no complications. Maternal ETI therapy likely led to meconium ileus resolution with evidence supporting the continued infant benefit of ETI therapy by breastfeeding [116]. All these cases highlight the critical need for additional data to guide the potential broader use of ETI therapy during the pre- and post-natal period to prevent or delay complications related to CF.

More than 95% of men with CF are infertile due to congenital bilateral absence of vas deferens [120]. Rotolo and collaborators evaluated seven men with CF that started to present testicular pain and discomfort within the first week after starting ETI therapy [117]. Physical and imaging analyses were normal, and the adverse effects were resolved within a week after onset, except for one individual that was diagnosed with epididymitis [117]. Further studies are needed to better understand the potential impact of ETI therapy on male fertility.

Taken together, these case reports suggest that ETI therapy may significantly reduce infertility/subfertility issues in women with CF. ETI therapy might also delay the appearance of disease signs in infants with CF via placental and breast milk transfer. Further studies are needed to understand the impact of ETI therapy on the embryonic development and infertility of men with CF.

### 3.7. Skin Implications

Skin-related adverse effects are commonly reported by PwCF after the start of ETI therapy. Several case reports have already reported rash, *Malassezia folliculitis*, and acne as common manifestations [121,122,123,124,125,126,127,128,129] (Table 8). Indeed, up to 10% of PwCF receiving ETI therapy were reported to present skin manifestations in clinical trials [17,18].

A case of pruritic rash was initially reported in a 24-year-old woman receiving ETI therapy [123] and then also observed in different case reports in adolescents with CF [124,128]. The manifestations appeared in the first week after the start of ETI therapy and were resolved soon after its discontinuation [124,128]. In another study, Diseroad and collaborators promoted ETI desensitization in two adolescents with CF, which were then able to restart ETI therapy [128]. Such an approach was performed due to the significant benefits in the respiratory symptoms promoted by ETI therapy [128], which is in agreement with other studies [121,131]. Bhaskaran and collaborators also reported a case of a 21-year-old woman who developed a rash 8 days after the start of ETI therapy [127]. In contrast to the other cases, she continued ETI therapy, due to the improvements in respiratory symptoms, and started to take antihistamines and emollient creams, which resulted in the cessation of skin manifestation after four weeks. She kept performing both therapies to prevent the reappearance of skin manifestations without the need for the use of corticosteroids [127].

Two cases of *Malassezia folliculitis* were reported by Pomi and collaborators [130]. They were women 19 and 24 years old, respectively, who presented popular rash. Malassezia was diagnosed by dermoscopic evaluation and treated with Fluconazole. In both cases, the ETI therapy was continued, and skin manifestations were resolved within one month of Fluconazole. Another skin manifestation commonly observed after the start of ETI therapy is the worsening of acne. Hudson and collaborators reported a case series of 18 PwCF who complained about the manifestations within 8 months after the start of ETI therapy [129]. Most individuals who present these manifestations are women, which might be related to hormonal profile and anticonception usage [18,129]. It has also been suggested that the alterations in salt concentration in sweat glands might alter the microbiome of the skin, as it changes in the lungs [132], but further studies are warranted in this field.

Taken together, these case reports suggest that, even though ETI therapy may result in skin-related adverse effects, these can be resolved by ETI desensitization or association with therapy for the skin issue. More studies are warranted to understand the effects of ETI therapy on the skin microbiome.

### 3.8. Other Implications

Miller and collaborators used the US National Research Databases, which have information on more than 30 million individuals, to assess the potential impact of ETI therapy on hospitalizations [133]. A total of 389 PwCF receiving ETI therapy were identified and compared with the outcomes of those who did not start any modulator therapy. Results indicated a significant decrease in the number of hospitalizations, infection-related visits, antibiotic use, and general hospital visits in PwCF receiving ETI therapy. Notably, antibiotic prescription and infection-related visits significantly dropped after the first weeks of the start of ETI therapy [133]. Walter and Bass used a different approach but with a similar aim [134]. They used electronic medical records and included data from 37 PwCF receiving ETI therapy, of which 16 presented advanced lung disease. The mean number of days per month of hospitalized PwCF decreased from 27 to four after the start of ETI therapy. Regarding the need for intravenous antibiotics, there was a decrease of more than 80% [134].

Digital clubbing is a feature commonly observed in PwCF and may be caused by the progression of lung or cardiac disease [135], which was demonstrated to be reversed in individuals who underwent lung transplantation [136]. Due to the multifaceted effects of ETI therapy, Mahlen and collaborators investigated whether this therapy has an impact on the clubbing index of 15 PwCF [137]. In parallel, similar measurements were performed in nine PwCF who underwent lung transplantation. Data from before and after three months of ETI therapy demonstrated a significant decrease in the clubbing index. A decrease in the clubbing index was also observed in lung transplant recipients, who were not receiving modulator therapy. Even though the underlying mechanisms of digital clubbing are not completely elucidated [135], these findings suggest a beneficial effect on lung disease aside from airflow and gas exchange [137].

Since long-chain ceramide concentrations were previously reported to be increased in PwCF and associated with disease severity [138], Westhölter and collaborators analyzed blood samples of PwCF before and 1 month after the start of ETI therapy to investigate lipid profile using liquid chromatography–mass spectrometry [139]. PwCF receiving ETI therapy demonstrated a significant improvement in several clinical outcomes (ppFEV_1_, BMI, and SCC). Meanwhile, a decrease in the concentration of various long-chain ceramides (C16Cer, C18Cer, C20Cer, and C24:1Cer) was observed in these individuals [139]. Similar findings were reported in two independent studies using bronchial epithelial cultures, thus demonstrating the ability of CFTR modulators in reversing lipid imbalance [140,141].

Various reports have been using qualitative approaches to assess the effects of ETI therapy on the well-being and quality of life of PwCF. DiMango and collaborators used CFQ-R to investigate therapeutic outcomes in PwCF after three months of ETI therapy. They found improvements not only in sinonasal symptoms but also in sleep and psychological symptoms [70]. Using the SNOT questionnaire, Bode and collaborators also reported improved scores in nasal, otologic, sleep, and emotional subdomains in children and adults with CF receiving ETI therapy [77]. Aspinall and collaborators used a different approach and interviewed 12 PwCF who were receiving ETI therapy for at least six months [142]. Overall, an improvement in self-relating quality of life in daily routine was observed. These include a reduction of coughing, more energy, an increase in appetite, and quality of sleep. The reduction in the frequency of exacerbation and the number of hospitalizations led these PwCF to feel healthier, which resulted in less anxiety. They also reported a feeling of more independence and hope [142].

Significant improvements in extrapulmonary symptoms were reported by Martin and collaborators in PwCF receiving ETI therapy [72]. By using online questionnaires, PwCF indicated feeling improvements in appetite, sleep quality, general well-being, physical self-esteem, and reduction of therapy burden, while there were no improvements related to gastrointestinal manifestations. Notably, the positive outcomes related to general well-being, such as improvement in confidence, physical appearance, and psychological issues, were evidenced soon after the start of ETI therapy. Many PwCF described indications of high therapy burden, symptom severity, depression, and a closed future marked by death or transplant before ETI therapy [72]. In another study, Welsner and collaborators used polysomnography methodology to investigate the impact of ETI therapy on the sleep of PwCF [143]. An improvement in objective sleep measures, such as oxygen saturation during sleep and apnea/hypopnea index, was observed, which may be associated with the increase in ppFEV_1_ and respiratory rates [143]. It is noteworthy that by increasing sleep quality, PwCF also observed an improvement in other aspects of their lives, including quality of life [142].

On the other hand, it has been suggested that ETI may cross the blood–brain barrier due to their lipophilic affinity and might contribute to some psychiatric and sleep disorders that have been reported after the start of the therapy [142,144,145,146]. Zhang and collaborators investigated the potential impact of ETI therapy on depression and anxiety of 100 PwCF [147]. Data collected included the nine-item self-report depression screening (PHQ-9) and seven-item self-report anxiety tolls (GAD-7). For those individuals who indicated depression and anxiety symptoms, psychotherapy and mental health interventions were also offered. Overall, no differences in PHQ-9 and GAD-7 were observed after the start of ETI therapy. Nevertheless, those individuals who required dose adjustment exhibited higher scores in the questionnaires. Moreover, 23% of the participants indicated sleeping issues after the start of ETI therapy [147].

Tindell and collaborators reported a case of a 19-year-old woman with CF who presented a previous history of mental conditions, such as anxiety, depression, and suicidality. After she started receiving ETI therapy, her mental disorders reappeared, requiring several adjustments in the doses of both ETI and psychiatric drugs, so she was able to continue both therapies without any further injury to her health [145]. In another study, Arslan and collaborators reported two cases of adolescents with CF who presented mental health issues soon after the start of ETI therapy [148]. In both cases, PHQ-9 was scored as moderate concern for depression, but there were also suicide attempts. Significant improvement in mental health symptoms was observed after the ETI dosage was reduced, which also decreased the score of the PHQ-9 questionnaire to minimal concern for depression [148]. Heo and collaborators also described six cases in which PwCF started to present mental health disorders within the first three months of ETI therapy [146]. The main symptoms reported were mental fogginess, memory issues, and word-finding difficulty, and different management was taken for each case in order to address these concerns. As it is known that PwCF have increased rates of developing depression and anxiety [149], a balance of benefits and potential risks of receiving ETI therapy with other drugs needs to be further discussed between the healthcare team and each individual with CF.

Another potential adverse effect has been reported by Gramegna and collaborators, who observed the onset of systemic arterial hypertension in four PwCF soon after the start of ETI therapy [150]. Even though they presented other CF-related complications, there was no previous risk of cardiovascular disease. After the start of ETI, they demonstrated an increase in systolic and diastolic arterial pressure levels, similar to Petersen and collaborators [96], which were then stabilized by anti-hypertensive therapy [150]. The reason for such adverse effects remains unclear, and further research is warranted.

## 4. Outlook and Concluding Remarks

**Figure 2:**
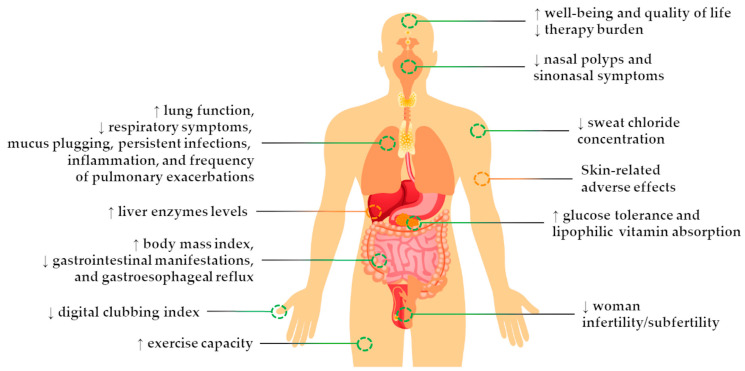
Major clinical effects of elexacaftor–tezacaftor–ivacaftor therapy in people with cystic fibrosis.

Major therapeutic progress has been achieved for PwCF over the last decade. The advent of CFTR modulator drugs into clinical practice constitutes a landmark in CF therapies. In particular, ETI therapy was approved by the FDA in late 2019 and by the EMA in early 2020 and represents a ‘highly effective’ therapy for the majority of PwCF worldwide. Various clinical studies have demonstrated that ETI therapy is associated with significant improvements in lung function, nutritional status, and respiratory and gastrointestinal symptoms not only in the short-term but also in the long-term (up to two years of follow-up to date) (Figure 2). However, further studies are needed to understand the impact of ETI therapy on drug–drug interaction profiles, eradication of different microbial infections, fertility/pregnancy, hospitalization, transplant, and mental health. Several adverse effects have also been reported, and close monitoring by a multidisciplinary healthcare team remains imperative. Since ETI therapy does not completely normalize the physiology of affected organs, nor reverse tissue remodeling, it is expected that an early start in life of ETI therapy could prevent severe tissue injury from occurring, which may thus result in much healthier and longer lives for PwCF.

Novel pre-clinical models are now available using samples from PwCF (i.e., bronchial/nasal epithelial cells and intestinal/respiratory organoids) to predict the magnitude of therapeutic responses at an individual level [12]. These tools are fundamental to expand the approval of ETI therapy for individuals carrying rare and ultra-rare CF genotypes that are responsive, providing thus a translational perspective in a precision medicine approach.

Despite these accomplishments, it is estimated that less than 15% of PwCF worldwide eligible for ETI therapy are actually receiving it [3], most likely due to the excessive cost [2,151,152]. Regulatory issues in national health systems also pose substantial challenges for the approval of novel therapies in different countries, and those should be optimized for life-changing therapies to reach PwCF in a more expedited way. In equivalent importance, not all PwCF are responsive to ETI therapy due to differences in the primary defects caused by CFTR mutations. Some PwCF may also decide not to adhere to this therapeutic regimen due to adverse effects. The CF therapeutic development landscape continues to expand, and it is expected that novel therapies will achieve the clinical stage in order to provide alternative options and more robust therapeutic outcomes for all PwCF.

## Figures and Tables

**Table 1:** Selected clinical trials assessing the safety and efficacy of IVA, LUMA-IVA, and TEZA-IVA in PwCF.

Study/Phase/Follow-Up	Study Population	Results (Primary Endpoints)
Ramsey et al., 2011 [13] STRIVE—Phase 3 48 weeks	161 PwCF carrying G551D-CFTR on at least one allele and ≥12 years old Subgroups: 83 with IVA (150 mg every 12 h), and 78 with placebo	Absolute change in ppFEV_1_ (week 24): +10.6 percentage points
Davies et al., 2013 [24] ENVISION—Phase 3 48 weeks	52 PwCF carrying G551D-CFTR on at least one allele and 6–11 years old Subgroups: 26 with IVA (150 mg every 12 h), and 26 with placebo	Absolute change in ppFEV_1_ (week 24): +12.5 percentage points
Davies et al., 2016 [25] KIWI—Phase 3 24 weeks	34 PwCF carrying at least one CFTR gating mutation and 2–5 years old Subgroups: 10 with IVA (50 mg every 12 h), and 24 with IVA (75 mg every 12 h)	Pharmacokinetics: Body weight was the most important predictor Safety: two children receiving IVA (50 mg every 12 h) experienced severe adverse effects
Wainwright et al., 2015 [14] TRAFFIC/TRANSPORT—Phase 3 24 weeks	1108 PwCF homozygous for F508del-CFTR and ≥12 years old Subgroups: 368 with LUMA (600 mg/day) + IVA (250 mg every 12 h), 369 with LUMA (400 mg every 12 h) + IVA (250 mg every 12 h), 371 with placebo	Absolute change in ppFEV_1_ (week 24): +3.3 percentage points for LUMA (600 mg/day) + IVA (250 mg every 12 h), and +2.8 percentage points for LUMA (400 mg every 12 h) + IVA (250 mg every 12 h)
Ratjen et al., 2017 [26] Phase 3 24 weeks	204 PwCF homozygous for F508del-CFTR and 6–11 years old Subgroups: 103 with LUMA (200 mg every 12 h) + IVA (250 mg every 12 h), and 101 with placebo	Average absolute change in LCI_2.5_ (week 24): −1.09 units for LUMA + IVA vs. placebo
Donaldson et al., 2018 [27] Phase 2 25 days of treatment + 25 days of washout (post-treatment observation period)	172 PwCF homozygous for F508del-CFTR and ≥18 years old Subgroups: 33 with TEZA (10 to 150 mg/day), 106 with TEZA (10 to 150 mg/day) + IVA (150 mg every 12 h), and 33 with placebo 18 PwCF heterozygous for F508del-CFTR and G551D-CFTR and ≥12 years old Subgroups: 14 with TEZA (100 mg/day) + IVA (150 mg every 12 h), and 4 with placebo (IVA monotherapy) (150 mg every 12 h)	Safety (day 56): the majority (81.4%) of adverse effects were mild to moderate Change in SCC (day 28): −6.04 mmol/L after treatment vs. placebo (homozygous for F508del-CFTR), −7.02 mmol/L after TEZA + IVA vs. IVA monotherapy (heterozygous for F508del-CFTR and G551D-CFTR)
Taylor-Cousar et al., 2017 [15] EVOLVE—Phase 3 24 weeks	475 PwCF homozygous for F508del-CFTR and ≥12 years old Subgroups: 235 with TEZA (100 mg/day) + IVA (150 mg every 12 h), and 240 with placebo	Absolute change in ppFEV_1_ (week 24): +4.0 percentage points
Rowe et al., 2017 [16] EXPAND—Phase 3 8 weeks of treatment +8 weeks of washout +8 weeks of treatment	481 PwCF heterozygous for F508del-CFTR and a residual-function CFTR mutation and ≥12 years old Subgroups: 162 with TEZA (100 mg/day) + IVA (150 mg every 12 h), 157 with IVA (150 mg every 12 h), and 162 with placebo	Absolute change in ppFEV_1_ (average of weeks 4 and 8): +6.8 percentage points for TEZA + IVA, +4.7 percentage points for IVA monotherapy

Abbreviations: CFTR—cystic fibrosis transmembrane conductance regulator; IVA—ivacaftor; LCI_2.5_—lung clearance index_2.5_; LUMA—lumacaftor; ppFEV_1_—percent predicted forced expiratory volume in one second; PwCF—people with cystic fibrosis; SCC—sweat chloride concentration; TEZA—tezacaftor.

**Table 2:** Clinical trials assessing the safety and efficacy of VX-659 or ELEXA in combination with TEZA plus IVA in PwCF.

Study/Phase/Follow-Up	Study Population	Results (Primary Endpoints)
Davies et al., 2018 [33] Phase 2 4 weeks	63 PwCF heterozygous for F508del-CFTR and an MF CFTR mutation and ≥18 years old Subgroups: 53 with VX-659 (80, 240, or 400 mg/day) + TEZA (100 mg/day) + IVA (150 mg every 12 h), and 10 with placebo 29 PwCF homozygous for F508del-CFTR and ≥18 years old Subgroups: 18 with VX-659 (400 mg/day) + TEZA (100 mg/day) + IVA (150 mg every 12 h), and 11 with placebo + TEZA (100 mg/day) + IVA (150 mg every 12 h) corresponding to the active control group	Safety and side-effects: Most adverse effects were mild or moderate Absolute change in ppFEV_1_ (day 29, heterozygous for F508del-CFTR and an MF CFTR mutation): +10.2 percentage points for VX-659 (80 mg/day), +12.0 percentage points for VX-659 (240 mg/day); +13.3 percentage points for VX-659 (400 mg/day) Absolute change in ppFEV_1_ (day 29, homozygous for F508del-CFTR): +9.7 percentage points
Keating et al., 2018 [34] Phase 2 4 weeks	65 PwCF heterozygous for F508del-CFTR and an MF CFTR mutation and ≥18 years old Subgroups: 53 with ELEXA (50, 100, or 200 mg/day) + TEZA (100 mg/day) + IVA (150 mg every 12 h), and 12 with triple placebo 28 PwCF homozygous for F508del-CFTR and ≥18 years old Subgroups: 21 with ELEXA (200 mg/day) + TEZA (100 mg/day) + IVA (150 mg every 12 h), and 7 with TEZA (100 mg/day) + IVA (150 mg every 12 h) corresponding to the active control group	Absolute change in ppFEV_1_ (week 4, heterozygous for F508del-CFTR and an MF CFTR mutation): +11.1 percentage points for ELEXA (50 mg/day), +7.9 percentage points for ELEXA (100 mg/day), +13.8 percentage points for ELEXA (200 mg/day) Absolute change in ppFEV_1_ (week 4, homozygous for F508del-CFTR): +11.0 percentage points for ELEXA (200 mg/day)
Heijerman et al., 2019 [17] Phase 3 4 weeks	107 PwCF homozygous for F508del-CFTR and ≥12 years old Subgroups: 55 with ELEXA (200 mg/day) + TEZA (200 mg/day) + IVA (150 mg every 12 h), and 52 with TEZA (100 mg/day) + IVA (150 mg every 12 h) corresponding to the active control group	Absolute change in ppFEV_1_ (week 4): +10.0 percentage points
Middleton et al., 2019 [18] Phase 3 24 weeks	107 PwCF heterozygous for F508del-CFTR and an MF CFTR mutation and ≥12 years old Subgroups: 200 with ELEXA (200 mg/day) + TEZA (100 mg/day) + IVA (150 mg every 12 h), and 203 with placebo	Absolute change in ppFEV_1_ (week 4): +13.8 percentage points
Zemanick et al., 2021 [35] Phase 3 24 weeks	29 PwCF homozygous for F508del-CFTR and 6–11 years old Subgroups: 16 (<30 kg) with ELEXA (100 mg/day) + TEZA (50 mg/day) + IVA (75 mg every 12 h), 13 (≥30 kg) with ELEXA (200 mg/day) + TEZA (100 mg/day) + IVA (150 mg every 12 h) 37 PwCF heterozygous for F508del-CFTR and an MF CFTR mutation and 6–11 years old Subgroups: 20 (<30 kg) with ELEXA (100 mg/day) + TEZA (50 mg/day) + IVA (75 mg every 12 h), 17 (≥30 kg) with ELEXA (200 mg/day) + TEZA (100 mg/day) + IVA (150 mg every 12 h)	Safety and tolerability: the majority (96.9%) of adverse effects were mild or moderate
Barry et al., 2021 [36] Phase 3 4-week run-in period 8 weeks	95 PwCF heterozygous for F508del-CFTR and a gating CFTR mutation and ≥12 years old Subgroups: 50 with ELEXA (200 mg/day) + TEZA (100 mg/day) + IVA (150 mg every 12 h), and 45 with IVA (150 mg every 12 h) corresponding to the active control group 163 PwCF heterozygous for F508del-CFTR and a residual-function CFTR mutation and ≥12 years old Subgroups: 82 with ELEXA (200 mg/day) + TEZA (100 mg/day) + IVA (150 mg every 12 h), and 81 with TEZA (100 mg/day) + IVA (150 mg every 12 h) corresponding to the active control group	Absolute change in ppFEV_1_ (week 8) from baseline (heterozygous for F508del-CFTR and a gating CFTR mutation): +5.8 percentage points Absolute change in ppFEV_1_ (week 8) from baseline (heterozygous for F508del-CFTR and an MF CFTR mutation): +2.5 percentage points
Griese et al., 2021 [37] Phase 3 24 weeks or longer	107 PwCF homozygous for F508del-CFTR and ≥12 years old Subgroups: ELEXA (200 mg/day) + TEZA (100 mg/day) + IVA (150 mg every 12 h) 399 PwCF heterozygous for F508del-CFTR and an MF CFTR mutation and ≥12 years old Subgroups: ELEXA (200 mg/day) + TEZA (100 mg/day) + IVA (150 mg every 12 h)	Safety: the majority of adverse effects were mild or moderate
Sutharsan et al., 2022 [38] Phase 3b 4-week run-in period 24 weeks	175 PwCF homozygous for F508del-CFTR, ≥12 years old, and with a baseline ppFEV_1_ = 40–90% Subgroups: 87 with ELEXA (200 mg/day) + TEZA (100 mg/day) + IVA (150 mg every 12 h), 88 with TEZA (100 mg/day) + IVA (150 mg every 12 h) corresponding to the active control group	Absolute change in CFQ-R respiratory domain score from baseline (week 24): +17.1 (14.1 to 20.1) points for the treatment group, and 15.9 (11.7 to 20.1) points between treatment and control group
Mall et al., 2022 [39] Phase 3b 24 weeks	121 PwCF heterozygous for F508del-CFTR and an MF CFTR mutation, 6–11 years old, and LCI_2.5_ ≥ 7.5 Subgroups: 39 (<30 kg) with ELEXA (100 mg/day) + TEZA (50 mg/day) + IVA (75 mg every 12 h), 21 (≥30 kg) with ELEXA (200 mg/day) + TEZA (100 mg/day) + IVA (150 mg every 12 h), and 61 with placebo	Absolute change in LCI_2.5_ (week 24): −2.29 (−2.60 to −1.97) units for ETI, and −2.26 (−2.71 to −1.81) units between treatment and placebo group
Nichols et al., 2022 [40] PROMISE—Post-approval study 6 months	487 PwCF aged ≥12 years with at least one allele of F508del-CFTR starting ETI therapy for the first time Subgroups (modulator use at baseline): 238 PwCF naïve to modulators, 34 PwCF using IVA, 215 PwCF using TEZA + IVA or LUMA + IVA	Absolute change in ppFEV_1_ from baseline (naïve to modulators): +10.8 percentage points Absolute change in ppFEV_1_ from baseline (IVA): +6.1 percentage points Absolute change in ppFEV_1_ from baseline (two-drug combination): +9.2 percentage points
Mayer-Hamblett et al., 2022 [41] SIMPLIFY 6 weeks	594 PwCF aged 12–17 years with ppFEV_1_ of 70% or more or those aged ≥18 years with ppFEV_1_ of 60% or more taking ETI and either or both mucoactive therapies (hypertonic saline or dornase alfa) for at least 90 days before screening Subgroups: 370 PwCF were randomly assigned to the hypertonic saline trial, and 477 were assigned to the dornase alfa trial. A subset of 253 participants participated in both trials	Absolute change in ppFEV_1_ in the hypertonic saline trial: −0.19% (−0.85 to 0.48) in the discontinuation group (n = 133) vs. 0.14% (−0.51 to 0.78) in the continuation group (n = 193) Absolute change in ppFEV_1_ in the dornase alfa trial: 0.18% (−0.38 to 0.74) in the discontinuation group (n = 199) vs. −0.16% (−0.73 to 0.41) in the continuation group (n = 193)

Abbreviations: CFQ-R—cystic fibrosis questionnaire-revised; CFTR—cystic fibrosis transmembrane conductance regulator; ELEXA—elexacaftor; ETI—elexacaftor–tezacaftor–ivacaftor; IVA—ivacaftor; LCI_2.5_—lung clearance index_2.5_; LUMA—lumacaftor; MF—minimal function; ppFEV_1_—percent predicted forced expiratory volume in one second; PwCF—people with cystic fibrosis; TEZA—tezacaftor.

**Table 3:** Clinical studies assessing the effects of ETI therapy in PwCF carrying at least one F508del-CFTR allele and advanced lung disease.

Study	Study Population	Main Results	Conclusion
Burgel et al., 2021 [43] Prospective, observational study	236 PwCF aged ≥12 years, with advanced lung disease and at least one copy of F508del-CFTR The study was performed between December 2019 and August 2020	Safety: no PwCF required discontinuation of ETI therapy, and the most prevalent adverse events were mild Effectiveness: Absolute increase in ppFEV_1_: +15.1; mean increase in weight: +4.2 kg; a significant decrease in the need for long-term oxygen and enteral tube feeding; lung transplantation: 11 of 15 patients that were waitlisted were taken off the candidate list	ETI therapy resulted in a rapid improvement in lung function even in PwCF with advanced lung disease
Carnovale et al., 2022 [44]	26 PwCF (F508del/F508del genotype) with advanced lung disease The study was performed between October 2019 and July 2020	Safety: No adverse events leading to discontinuation of ETI therapy were reported Effectiveness: The mean absolute improvement in ppFEV_1_ was 14.48 after 48 weeks. The mean absolute increase in BMI was 2.08. CFQ-R respiratory domain score improved by 32.6 points from baseline after 48 weeks	ETI therapy was safe and effective in PwCF homozygous for F508del-CFTR and advanced lung disease
O’Shea et al., 2020 [45] Observational study	14 PwCF (F508del/F508del or F508del/MF genotypes) with advanced lung disease The study was performed between December 2019 and July 2020	Safety: ETI therapy was well-tolerated Effectiveness: Improvement in ppFEV_1_, SCC, BMI, and infective exacerbations requiring hospitalization	ETI therapy improved multiple outcome measures for PwCF with advanced lung disease
Terlizzi et al., 2021 [46] Retrospective, observational study	Three adults with CF (F508del/unknown genotype) with advanced lung disease The study was performed between October 2019 and April 2021	Safety: No adverse events led to the discontinuation of ETI therapy Effectiveness: SCC decreased progressively in all patients; relevant improvements of ppFEV_1_; all individuals discontinued oxygen therapy after 4–12 weeks of ETI therapy as well as reduced the need for antibiotic therapy	ETI was a feasible therapy for PwCF with F508del/unknown genotype
Carnovale et al., 2021 [47] Retrospective cohort study	47 PwCF (F508del/MF genotypes) with ppFEV_1_ < 40% or who were considered for lung transplantation The study was performed between October 2019 and May 2020	Safety: No treatment-related adverse events leading to discontinuation were reported Effectiveness: Improvements in ppFEV_1_ and 6-min walking test (m) distance were reported, and a significant reduction in the rate of pulmonary exacerbations and the need for oral and intravenous antibiotic therapy	ETI was a safe and effective therapy for PwCF with F508del/MF genotype and advanced pulmonary disease
Salvatore et al., 2021 [48] Case report	A 50-year-old woman with CF (F508del/G1244E genotype) with advanced lung disease	Safety: No ETI therapy-related adverse events were reported Effectiveness: Improvements in ppFEV_1_, SCC, BMI, and CFQ-R respiratory domain scores were reported	ETI therapy may have therapeutic potential for PwCF with advanced lung disease, particularly for whom lung transplantation is contraindicated
Gur et al., 2021 [49] Case report	A 24-year-old woman with CF (F508del/R1066C genotype) with severe progressive *Mycobacterium abscessus* lung disease and nutritional failure	Effectiveness: After 1 year of treatment, ppFEV1 improved from 26% to 45%, BMI improved from 16.4 to 21, and the sputum cultures were negative since the start of ETI	ETI treatment may open a new horizon in continuous efforts to overcome persistent infections in PwCF
Migliorisi et al., 2022 [50] Case-Control study	26 PwCF with at least one F508del mutation and ppFEV_1_ ≤ 40%	Effectiveness: After 1 year of treatment, respiratory, pancreatic, and sweat function, BMI, and quality of life improved in the case group patients; the rate of airway infections and pulmonary exacerbations were reduced; sputum samples collected progressively resulted in no detection of relevant pathogenic microorganisms	Long-term treatment with ETI can give rise to changes in pulmonary microbiota and may reduce the need for antibiotics
Macconi et al., 2022 [51] Prospective, observational study	3 PwCF with genotypes F508del/N1303K, F508del/R553Q, and F508del L065P with advanced lung disease	Effectiveness: MRI performed 1 month before and 3 months after the start of ETI therapy showed a significant reduction in mucus plugging and bronchial wall thickening	Chest MRI could be a useful tool to evaluate disease progression in PwCF
Stylemans et al., 2022 [52] Real-life follow-up study	14 adults with CF (F508del/F508del and F508del/MF genotypes) The study was performed between December 2019 and November 2020	Safety: ETI therapy was well-tolerated; one patient had to interrupt treatment due to liver injury Effectiveness: Marked improvements in peripheral lung function	Marked effects on ppFEV_1_ and pulmonary exacerbations could be obtained in real life under ETI therapy, even in severely obstructive PwCF
Kos et al., 2022 [53] Longitudinal, real-life, observational study	19 PwCF with at least one F508del-CFTR allele and advanced lung disease	Safety: ETI therapy was well-tolerated, and only mild adverse effects were reported Effectiveness: BMI and mean absolute FEV_1_ improved; there was a marked reduction in the frequency of pulmonary exacerbations	Clinical benefits of ETI therapy were maintained for 12 months in PwCF with advanced lung disease
Salvatore et al., 2022 [54] Observational study	Nine children with CF aged 6–11 years with at least one copy of F508del-CFTR and advanced lung disease	Safety: ETI therapy was safe and there was no need for treatment discontinuation Effectiveness: After 24 weeks, the mean absolute changes in ppFEV_1_, BMI, and SCC were +22.4 points, +0.60 kg/m^2^, and −79.2 mmol/L, respectively; the CFQ-R respiratory domain score increased from the median baseline score of 25 to 100 after 24 weeks; treatment led to a reduction of the rate of antibiotic treatment to 80% over the 24 weeks of the study	ETI therapy improved lung function, nutrition status, and quality of life of children with CF aged 6–11 years with advanced lung disease and at least one F508del-CFTR allele
Dhote et al., 2023 [55] Prospective study	79 adults with CF carrying at least one F508del-CFTR allele and advanced lung disease The study was performed between December 2019 and July 2021	Effectiveness: 12 months of ETI therapy was associated with a significant decrease in circulating neutrophils, monocytes, and platelets, but not lymphocytes, to values within the laboratory reference range	ETI therapy may normalize neutrophil counts in PwCF with advanced lung disease
McCoy et al., 2023 [56] Retrospective study	18 PwCF with at least one F508del-CFTR allele and advanced lung disease The study was performed between July 2019 and September 2022	Safety: ETI was well-tolerated, without the need for treatment discontinuation Effectiveness: After 24 months, SCC decreased from 84–140 mmol/L to 15–63 mmol/L; ppFEV_1_ improved from a median of 27.5 to 45.0; and BMI significantly increased from 19.1 kg/m^2^ to 22.8 kg/m^2^	ETI was safe with positive changes in nutrition and respiratory symptoms, CFQ-R, and lung function, and a reduction in therapy burden was maintained for 2 years after the start of ETI therapy in PwCF with advanced lung disease

Abbreviations: BMI—body mass index; CF—cystic fibrosis; CFQ-R—cystic fibrosis questionnaire-revised; CFTR—cystic fibrosis transmembrane conductance regulator; ETI—elexacaftor-tezacaftor-ivacaftor; MF—minimal function; MRI—magnetic resonance imaging; ppFEV_1_—percent predicted forced expiratory volume in one second; PwCF—people with cystic fibrosis; SCC—sweat chloride concentration.

**Table 4:** Clinical studies assessing the effects of ETI therapy on respiratory symptoms and signs in PwCF.

Study	Study Population	Main Results	Conclusion
Wucherpfenning et al., 2022 [64] Retrospective, observational study	43 adults with CF and at least one F508del-CFTR allele The study was performed between June 2020 and August 2021	Effectiveness: MRI revealed improvements in chest MRI morphology score and chronic rhinosinusitis–MRI scores after the start of ETI therapy	MRI studies indicated reversibility of structural lung and paranasal sinus abnormalities in PwCF who received ETI therapy. These improvements correlated well with proportionally improved spirometry
Graeber et al., 2022 [65] Prospective, observational study	91 PwCF aged ≥12 years; 46 homozygous for F508del-CFTR and 45 heterozygous (F508del/MF genotype)	Effectiveness: In individuals homozygous for F508del-CFTR, LCI improved by 15.3%, and MRI global improved by 29.3% after the start of ETI therapy In individuals heterozygous (F508del/MF), ETI therapy led to improvements of LCI of 23.4% from baseline and MRI global of 25.6% from baseline	ETI therapy improved lung ventilation and abnormalities in lung morphology in PwCF with at least one copy of F508del-CFTR in a real-world setting
Streibel et al., 2023 [66] Retrospective, observational study	24 children with CF	Effectiveness: Evaluation of structural and functional MRI parameters before and two weeks after the start of ETI therapy showed a significant improvement in lung function	Functional and structural MRI are promising tools to complement information obtained using lung function measures (spirometry and LCI)
Stapleton et al., 2022 [67]	34 PwCF with ≥12 years old (28 participants completed both study visits) The study was performed between November 2019 and March 2020	Effectiveness: Sinonasal symptoms improved rapidly by day 7 of ETI therapy, and the improvement was persistent for up to 189 days	ETI therapy improved the sinonasal quality of life scores, nasal endoscopy scores, and sinonasal CT scans and led to the regression of nasal polyps
Lee et al., 2022 [68] A score-matched historical cohort study	Total of 468 PwCF treated with ETI (n *=* 367 F508del/MF genotype; n *=* 101 homozygous for F508del) Total of 1714 PwCF untreated (control group) (n *=* 1242 F508del/MF genotype; n *=* 472 homozygous for F508del)	Effectiveness: Participants treated with ETI had, on average, no loss of pulmonary function over 2 years in comparison with a decline in ppFEV_1_ of 1.92% annually in untreated controls	ETI therapy showed sustained lung function for an extended period
Bec et al., 2022 [69] Retrospective, observational study	12 adults with CF homozygous or heterozygous for F508del-CFTR The study was performed between April 2018 and November 2021	Effectiveness: After one year, ETI therapy showed improvement in lung damage on chest CT and a decrease in the visual Brody-II score of 21% due to lower mucus plugging and peribronchial thickening	The study supports the start of ETI therapy early in life to avoid lung sequelae
DiMango et al., 2021 [70] Observational study	43 adults with CF with at least one F508del-CFTR allele; 23 participants were taking a CFTR modulator at the time of participation	Effectiveness: ETI therapy significantly improved both SNOT and CFQ-R scores at 3 months	PwCF who had been taking other CFTR modulators before the start of ETI therapy had more pronounced benefits (higher SNOT score at baseline) compared to those who were not taking any CFTR modulators
Causer et al., 2022 [71] Case series	Three adolescents with CF homozygous for F508del-CFTR	Effectiveness: ETI therapy improved exercise capacity with a VO_2_ peak observed in all three cases (+17.65%, +52.4%, and +32.9%, respectively)	ETI therapy may improve exercise capacity in PwCF
Martin et al., 2021 [72] Mixed method study through an online questionnaire	101 PwCF aged 12 years or older with advanced lung disease receiving ETI The online questionnaire was available from July to August 2020	Effectiveness: Participants reported a rapid impact on sleep quality, general well-being, respiratory symptoms, and a reduction in treatment burden	After the start of ETI therapy, PwCF reported rapid and positive physical, psychological, and social effects with an improvement in quality of life
Bacon et al., 2022 [73] Observational study	34 PwCF aged ≥12 years (28 participants completed the study) The study was performed between November 2019 to March 2020	Effectiveness: There was no significant difference in the olfactory test (UPSIT) after the start of ETI therapy	Larger studies and longer follow-up periods are needed given that the small cohort did not show improvement in UPSIT score
Beswick et al., 2022 [74] Prospective, observational study	25 adults with CF (F508del/F508del and F508del/MF genotypes) and with chronic rhinosinusitis The study was performed between August 2019 to October 2020	Effectiveness: After 6 months of follow-up, sinus CT opacification improved by a mean of 22.9% with ETI therapy	ETI therapy was associated with clinical improvements in sinus disease; however, it did not fully resolve sinus disease after 6 months of treatment
Castellanos et al., 2022 [75] Observational study	23 children with CF	Effectiveness: ETI therapy improved sinonasal symptoms and CFQ-R score	ETI therapy was associated with an improvement in sinonasal function and quality of life in children with CF
Sheikh et al., 2022 [76] Observational study	48 adults with CF carrying at least one F508del-CFTR allele and 20 healthy adult controls	Effectiveness: ETI therapy improved clinical outcomes (ppFEV_1_ and BMI), reduced bacterial infection in respiratory cultures, and significantly reduced circulating neutrophils and levels of pro-inflammatory cytokines (IL-6, IL-8, and IL-17A)	ETI therapy may reduce neutrophilic inflammation and neutrophil-mediated lung disease
Bode et al., 2023 [77] A cross-sectional, retrospective study	43 PwCF (six children) who started ETI therapy and 20 controls (PwCF naïve to modulator therapy)	Effectiveness: Overall, a reduction in the SNOT-22 score and objective clinical improvement were observed in the PwCF receiving ETI therapy	ETI may promote clinical benefits on upper airway symptoms in PwCF
Giallongo et al., 2023 [78] Retrospective study	Nine PwCF aged ≥12 years and ppFEV_1_ < 40%	Effectiveness: After 12 months of ETI therapy, the absolute change from baseline in nocturnal cardiorespiratory polygraphy parameters showed a significant improvement in nocturnal oxygenation, time spent with SpO_2_ ≤ 90%, and respiratory rate	ETI therapy resulted in improved nocturnal SpO_2_ at month 3 that was sustained up to 12 months after the start of ETI
Beck et al., 2023 [79] Retrospective study	124 PwCF aged ≥12 years The study was performed between October 2019 and October 2021	Effectiveness: Culture positivity for *Pseudomonas aeruginosa*, methicillin-resistant, and methicillin-susceptible *Staphylococcus aureus* was 54%, 33%, and 31%, respectively, before the start of ETI therapy; the prevalence of detection decreased to 30%, 32%, and 24%, respectively	ETI therapy results in a significant reduction in the detection of common bacterial pathogens in CF respiratory cultures, after 12 months of treatment

Abbreviations: BMI—body mass index; CF—cystic fibrosis; CFQ-R—cystic fibrosis questionnaire-revised; CFTR—cystic fibrosis transmembrane conductance regulator; CT—computerized tomography; ETI—elexacaftor–tezacaftor–ivacaftor; IL—interleukin; LCI—lung clearance index; MF—minimal function; MRI—magnetic resonance imaging; ppFEV_1_—percent predicted forced expiratory volume in one second; PwCF—people with cystic fibrosis; SNOT—sino-nasal outcome test; SpO_2_—oxygen saturation; UPSIT—University of Pennsylvania Smell Identification Test.

**Table 5:** Clinical studies assessing the effects of ETI therapy in PwCF referred to the transplant of post-transplant recipients.

Study	Study Population	Main Results	Conclusion
Bermingham et al., 2021 [87] Retrospective study	50 adults with CF with advanced lung disease	Safety: ETI therapy was well-tolerated, and no participant required discontinuation Effectiveness: 64% of patients experienced an improvement of ≥5% in absolute ppFEV_1_	Clinical improvements after ETI therapy resulted in adjustments to lung transplantation planning using CFF guidelines
Martin et al., 2022 [88] Prospective observational study	65 PwCF with advanced lung disease (lung transplant candidates at the time of starting ETI therapy)	Safety: Adverse events were mild and transient Effectiveness: Most participants experienced rapid and clinically meaningful improvements in lung function, pulmonary exacerbations, gas exchange, and nutritional status	ETI therapy improved multiple outcome measures for PwCF with advanced lung disease
Benniger et al., 2021 [89] Retrospective observational study	9 PwCF, homozygous for F508del, who underwent bilateral lung transplantation	Effectiveness: BMI, sinus, and GI symptoms improved after the start of ETI therapy	ETI therapy did not cause graft function decline or significant impact on immunosuppressive drug regimens or doses, in post-transplant recipients
Doligalski et al., 2022 [90] Observational study	13 PwCF with potential benefits for lung transplant recipients and at least one copy of F508del-CFTR The study was performed between November 2019 and July 2021	Safety: Five participants discontinued therapy due to declining pulmonary function, mood disturbances, or lack of benefit; four participants reported adverse events; and three interrupted treatment temporarily Effectiveness: Six participants reported improvement in sinus symptoms and four reported improvement in gastrointestinal symptoms; the tacrolimus dose declined by 50% after the start of ETI therapy	ETI therapy was poorly tolerated and showed modest extra-pulmonary benefit
Ramos et al., 2022 [91] Observational study	94 PwCF who started ETI therapy after a lung transplant	Safety: ≥40% of participants stopped ETI therapy due to adverse events or lack of perceived benefit Effectiveness: Frequency of antibiotic prescriptions decreased, hemoglobin A1c improved, BMI did not show a significant difference	The risks and benefits of ETI therapy after a lung transplant should be further determined in a greater CF population with a lung transplant
McKinzie et al., 2022 [92] Case series	Two PwCF who had a liver transplant: Subject 1— F508del/Nt1652delCTT; Subject 2— F508del/F508del	Safety: Neither individual required dose adjustment in their baseline immunosuppression regimens: Subject 1—significant elevations in liver function tests were drug-associated; after dose correction, liver profiles remain acceptable Subject 2—Mild acute cellular rejection episode that was successfully treated with corticosteroids Effectiveness: Improvement in lung function and nutritional status	There may be a role for ETI therapy in liver transplant recipients with close monitoring for adverse effects
Ragan et al., 2022 [93] Retrospective case series	10 PwCF who had liver transplant: Subject 1—F508del/F508del Subject 2—F508del/W1282X Subject 3—F508del/F508del Subject 4—F508del/F508del Subject 5—F508del/F508del Subject 6—F508del/F508del-Y301C Subject 7—F508del/W1282X Subject 8—F508del/F508del Subject 9—F508del/Q493X Subject 10—F508del/1216+1G->A	Safety and Effectiveness of ETI therapy: Subject 1—no significant adverse events were observed; quality of life improved Subject 2—treatment was tolerated with no marked adverse events; quality of life improved from baseline Subject 3—individuals had abdominal pain with evidence of drug-induced liver disease; therapy was discontinued Subject 4—individuals had symptoms of Tacrolimus toxicity, namely severe gastrointestinal onset, and acute kidney injury; therapy was discontinued Subject 5—there was no evidence of drug-induced liver injury; quality of life improved Subject 6—there was no evidence of drug-induced adverse events; respiratory symptoms improved Subject 7—treatment was tolerated with no marked adverse events; ppFEV_1_ increased to 100% after 2 weeks Subject 8—treatment was tolerated after some drugs dose adjustments; improvement in symptoms and quality of life Subject 9—there was no evidence of drug-induced adverse events; respiratory symptoms and quality of life improved Subject 10—treatment was tolerated after the reduction of tacrolimus dose; respiratory symptoms and quality of life improved	PwCF who underwent to liver transplant may initiate ETI therapy with a close therapeutic drug monitoring of immunosuppression
Ørum et al., 2022 [94]	Four PwCF who had solid organ transplants: Two had a bilateral lung transplant and two had a liver transplant	Safety: ETI therapy was well-tolerated with no adverse events that led to discontinuation Effectiveness: pulmonary symptoms improved in all subjects but the increase in FEV_1_ was significantly larger in liver transplant recipients with native lungs	All participants experienced subjective pulmonary and extra-pulmonary improvements after the start of ETI therapy

Abbreviations: BMI—body mass index; CF—cystic fibrosis; ETI—elexacaftor–tezacaftor–ivacaftor; ppFEV_1_—percent predicted forced expiratory volume in one second; GI—gastrointestinal; PwCF—people with cystic fibrosis.

**Table 6:** Clinical studies assessing the effects of ETI therapy on gastrointestinal symptoms and signs in PwCF.

Study	Study Population	Main Results	Conclusion
Schwarzenberg et al., 2022 [95] PROMISE Prospective observational study	438 PwCF aged ≤ 12 years and at least one F508del-CFTR allele	Effectiveness: Evaluation of GI symptoms using validated questionnaires (PAGI-SYM, PAC-SYM, and PAC-QOL) showed improvement; the fecal marker of inflammation (calprotectin) decreased, while pancreatic insufficiency measured by fecal elastase did not improve	After 6 months of ETI therapy, changes in GI symptoms were clinically unimportant, emphasizing the need for continued attention to GI disease
Petersen et al., 2021 [96] Retrospective observational study	134 adults with CF and at least one F508del-CFTR allele	Effectiveness: BMI substantially increased, and systolic and diastolic blood pressure also increased; in individuals with CFRD, total cholesterol, LDL-c, and HDL-c increased; in individuals without CFRD, random blood glucose, and hemoglobin A1c decreased	PwCF receiving ETI therapy should be monitored for evidence of overnutrition and related complications, such as hypertension and hypercholesterolemia
Scully et al., 2021 [97] Prospective observational study	33 adults with CF and at least one F508del-CFTR allele	Effectiveness: The start of ETI therapy was associated with significant improvements in continuous glucose monitoring-derived measures of average glucose, hyperglycemia, and glycemic variability	ETI therapy in adults with CF was associated with improvement in continuous glucose monitoring-derived measures of hyperglycemia and glycemic variability with no effect on hypoglycemia
Wright et al., 2021 [98] Retrospective observational study	76 PwCF with at least one F508del-CFTR allele	Effectiveness: ETI therapy increased vitamin D absorption as measured by serum 25-hydroxyvitamin D	The increase in serum 25-hydroxyvitamin D may reduce the dose of cholecalciferol and/or the need for multivitamin products
Shakir et al., 2022 [99] Prospective observational study	32 PwCF with at least one F508del-CFTR allele and advanced lung disease	Effectiveness: Sinonasal symptoms, gastroesophageal reflux, and extraesophageal reflux improved substantially within three months after the start of ETI therapy	ETI therapy improved upper GI symptoms, lung function, BMI, and quality of life in PwCF with advanced lung disease
Safirstein et al., 2020 [100] Case series	Seven adults with CF with different CF genotypes who developed biliary colic after the start of ETI therapy	The approximate number of days on ETI therapy before biliary symptoms and pathology report findings post-cholecystectomy: Subject 1—27 days, chronic cholecystitis with cholelithiasis and serosal fibrous adhesions; Subject 2—1 day, acute cholecystitis with mucosal necrosis and cholelithiasis; Subject 3—3 days, chronic cholecystitis with cholelithiasis, extensive mucosal erosion, and wall fibrosis and serositis; Subject 4—1 day, chronic cholecystitis with cholelithiasis; Subject 5—one day before treatment, not performed; Subject 6—14 days, acute cholecystitis with cholelithiasis with obstruction of the neck; Subject 7—1 day, chronic cholecystitis with cholelithiasis.	Asymptomatic gallbladder disease may be exacerbated after the start of ETI therapy in adults with CF
Lowry et al., 2021 [101] Case report	One individual with CF (F508del/c4077-4080delTGTTinsAA) with no known history of liver disease	Safety: Transaminase values increased after 5 months of ETI therapy; liver enzyme values normalized after 6 months of ETI therapy discontinuation; other identified sources of injury were excluded Effectiveness: Over ETI therapy, ppFEV_1_ improved from a baseline of 35% predicted to 53% predicted	Attempts to prevent liver injury will be important for the long-term use of CFTR modulators
Korten et al., 2022 [102] Observational study	16 adolescents with CF with at least one F508del-CFTR allele	Effectiveness: Glucose tolerance measured by oral glucose tolerance tests improved after the start of ETI therapy	ETI therapy shows beneficial effects on endocrine pancreatic function
Chan et al., 2022 [103] Prospective study	20 PwCF with at least one F508del-CFTR allele	Effectiveness: BMI z-score, insulin secretion, and resistance increased; no significant differences were observed in glucose tolerance and β-cell function	One year after the start of ETI therapy, insulin secretion increased but insulin resistance also increased, and there was no significant change in β-cell function
Mainz et al., 2022 [104]	107 PwCF with at least one F508del-CFTR allele	Effectiveness: Abdominal symptoms before and during ETI therapy were assessed using CFAbd-Score: the mean total CFAbd-Score decreased significantly by 29% during therapy	ETI therapy results in a significant reduction of abdominal symptoms
Steinack et al., 2023 [105] Retrospective, observational study	33 PwCF carrying at least one F508del CFTR allele	Effectiveness: At least 3 months after the start of ETI therapy, 48.5% of PwCF improved their glucose tolerance, 39.4% remained unchanged and 12.1% deteriorated	ETI therapy likely improves glucose tolerance without increasing insulin secretion
Tewkesbury et al., 2023 [106]	255 PwCF (30.6% had a diagnosis of CF-related liver disease)	Safety: Overall, ETI therapy led to a significant rise in AST, ALT, and bilirubin values at 3 months (median values remained in the normal range), which was sustained at 12 months with no further significant increase; no differences were observed between PwCF with or without liver disease	Pre-existing CF-related liver disease does not appear to correlate with alterations in liver tests and should not prevent the start of ETI therapy
Francalanci et al., 2023 [107] Cross-section, retrospective study	318 PwCF (3 children aged <2 years, 135 children aged 2–18 years, and 180 adults)	Effectiveness: One year of ETI therapy increased BMI and the levels of all circulating fat-soluble vitamins more consistently compared with previous modulators	ETI therapy promotes a beneficial effect on nutritional status and circulating levels of fat-soluble vitamins
Schnell et al. 2023 [108] Prospective observational study	20 PwCF (10 aged < 20 years) The study was performed between September 2020 and November 2021	Safety: In PwCF < 20 years, ARFI SWV increased after 6 months of ETI therapy; bile acid profiles revealed an increase in glycine-conjugated derivatives; there was a positive correlation between ARFI SWV values and glycine-conjugated derivatives	ETI therapy may lead to an early increase in liver stiffness and altered bile metabolism in children with CF. Evaluation of bile acid profile and liver stiffness can be useful tools to monitor hepatic alterations
Wood et al., 2023 [109] Retrospective study	83 adult PwCF	Safety: Transaminase elevations of >3 times the upper limit of normal were experienced by 11% of PwCF and an elevation of ≥25% above baseline was experienced by 75% of participants, during ETI therapy	Transaminase value elevation is a common feature in PwCF receiving ETI therapy.

Abbreviations: ALT—alanine aminotransferase; ARFI SWV—share wave velocity derived by acoustic radiation force impulse imaging; AST—aspartate aminotransferase; BMI—body mass index; CF—cystic fibrosis; CFTR—cystic fibrosis transmembrane conductance regulator; ETI—elexacaftor–tezacaftor–ivacaftor; GI—gastrointestinal; PAC-SYM—Patient Assessment of Constipation-Symptoms; PAC-QOL—Patient Assessment of Constipation-Quality of Life; PAGI-SYM—Patient Assessment of Upper Gastrointestinal Disorders-Symptom; PwCF—people with cystic fibrosis.

**Table 7:** Clinical studies assessing the effects of ETI therapy on fertility and pregnancy in PwCF.

Study	Study Population	Main Results	Conclusion
O’Connor et al., 2021 [111] Case series	14 women with CF with childbearing potential The study was performed between October 2019 and May 2020	14 women got pregnant 8 weeks after the start of ETI therapy; 7 individuals were attempting conception and had a history of subfertility/infertility; 7 participants were not attempting to conceive, using methods of contraception	There is a need for further studies to clarify the reproductive implications of ETI therapy and women’s pre-conception counseling
Chamagne et al., 2022 [112] Case study	A 32-year-old woman with CF, homozygous for F508del-CFTR	During the first pregnancy, she was not using ETI therapy and she got pregnant after 7 years of different in vitro fertilization cycles; in the second pregnancy, she was receiving ETI therapy for 20 days and became spontaneously pregnant	This case report shows a real benefit for the mother without the detection of any adverse fetal outcomes
Collins et al., 2022 [113] Observational study	3 women with CF, homozygous for F508del-CFTR, and their infants	Results demonstrated a relatively high concentration of ETI in cord blood and low levels in breast milk and infant blood	These findings suggest that ETI can cross the placenta leading to fetal exposure in women continuing ETI therapy during pregnancy
Fortner et al., 2021 [114] Case report	A woman with CF, heterozygous for F508del-CFTR	Six weeks after starting ETI therapy, she became pregnant; an infant with CF (F508del/F508del genotype) was born without any apparent organ dysfunction and the follow-up revealed high SCC	For this infant, exposure to ETI therapy in utero and through maternal breastmilk may have helped delay organ damage
Balmpouzis et al., 2022 [115] Case report	A 30-year-old woman with CF (F508del/Y1092X genotype) and advanced lung disease	Four months after the start of ETI therapy, an unplanned pregnancy occurred; no complications occurred up to the 31st week of pregnancy when she presented symptoms of threatened preterm labor and a rhinovirus infection; after childbirth, oxygen therapy was stopped; no further complications were reported over the one-year follow-up	ETI therapy has changed family planning and childbearing decisions for PwCF
Szentpetery et al., 2022 [116]	A woman (F508del carrier) pregnant with a fetus with CF (F508del/F508del genotype)	The mother started to receive ETI therapy to resolve bowel dilation in the fetus	Maternal ETI therapy likely resulted in the resolution of meconium ileus
Rotolo et al., 2020 [117] Case series	Seven men with CF and at least one F508del-CFTR allele	Individuals reported testicular pain or discomfort within the first two weeks of ETI therapy	Further study is required to determine the impact of this therapy on male fertility

Abbreviations: CF—cystic fibrosis; CFTR—cystic fibrosis transmembrane conductance regulator; ETI—elexacaftor–tezacaftor–ivacaftor; SCC—sweat chloride concentration.

**Table 8:** Clinical studies assessing the effects of ETI therapy on skin symptoms and signs in PwCF.

Study	Study Population	Main Results	Conclusion
Stashower et al., 2021 [123] Case report	24-year-old woman	One week after the start of ETI therapy, she presented with blanching, annular, pruritic plaques with dusky centers, and swelling in the hands, feet, and face	She cleared within one week of discontinuation of ETI therapy, suggesting the diagnosis of urticaria multiforme secondary to ETI
Goldberg et al., 2021 [124]	12-year-old boy (F508del/R347P)	One week after the start of ETI therapy, he presented with a rash consisting of pink-to-erythematous, edematous, round papules coalescing into plaques	Based on the clinical and histologic features, a diagnosis of Urticaria multiforme-like drug eruption due to ETI therapy was made
Bhaskaran et al., 2022 [127]	21-year-old woman (F508del/F508del)	Eight days after the start of ETI therapy, she developed a maculopapular rash that was violaceous and red over her shins, progressing to her thighs; the rash worsened in the next 36 h to progress centripetally affecting her back, anterior chest wall, anterior abdominal wall, neck, and arms	She needed supportive therapy for pruritus but never used steroids; the rash resolved spontaneously without stopping ETI therapy after four weeks, leaving no residual scars or discoloration
Diseroad et al., 2022 [128]	Subject 1—12-year-old girl (F508del/5T-M470V) Subject 2—14-year-old boy (Y569D/Y569D)	Subject 1—She developed a pruritic, maculopapular rash on her lower back, eyelids, ears, knees, hands, and head 30 min after the first dose of ETI that progressively worsened throughout the day Case 2—Eight days after starting ETI, he developed a pruritic, maculopapular rash on his hands that spread to his feet, palms, knee, and face	Both subjects completed ETI rechallenge with a practical titration schedule; the use of antihistamines with or without a short course of glucocorticosteroids might have aided in the resolution of the rash
Hudson et al., 2022 [129]	18 PwCF ranging from 19 to 38 years old	18 individuals reported either new or worsening acne symptoms within 8 months of ETI therapy, with 9 individuals reporting this manifestation within the first 3 months of ETI therapy	The underlying mechanism responsible for this adverse effect remains unknown
Pomi et al., 2022 [130]	Subject 1—a 19-year-old woman (F508del/F508del) Subject 2—a 24-year-old woman	Subject 1—Three days after starting ETI, she developed a widespread follicular papulopustular itchy rash localized to the abdomen and upper limbs progressively involving the buttocks and lower limbs; *Malassezia folliculitis* developed after ten days from re-administration of ETI Subject 2—An itchy papulopustular rash located on the back was observed 9 days after starting ETI, and the presence of Malassezia in the follicular pustule was confirmed	ETI therapy may induce changes in the skin microbiome, potentially able to favor colonization and proliferation of *Malassezia sp*; given the therapeutic benefit of ETI and its increasing use, oral desensitization may be considered as an option to avoid the full discontinuation of this therapy

Abbreviations: ETI—elexacaftor–tezacaftor–ivacaftor; PwCF—people with cystic fibrosis.

## Abbreviation

BMI—body mass index; cAMP—cyclic adenosine monophosphate; CF—cystic fibrosis; CFQ-R—CF questionnaire revised; CFRD—CF-related diabetes; CFTR—CF transmembrane conductance regulator; CT—computed tomography; ELEXA—elexacaftor; EMA—European Medicines Agency; ETI—elexacaftor–tezacaftor–ivacaftor; FDA—Food and Drug Administration; GAD-7—general anxiety disorder-7; GI—gastrointestinal; IL—interleukin; IVA—ivacaftor; LCI—lung clearance index; LUMA—lumacaftor; MF—minimal function; MRI—magnetic resonance imaging; ppFEV_1_—percent predicted forced expiratory volume in one second; PHQ-9—patient health questionnaire-9; PwCF—people with CF; SCC—sweat chloride concentration; SNOT—sino-nasal outcome test; TEZA—tezacaftor.
